# Biomimetic bone-periosteum scaffold for spatiotemporal regulated innervated bone regeneration and therapy of osteosarcoma

**DOI:** 10.1186/s12951-024-02430-7

**Published:** 2024-05-15

**Authors:** Yan Xu, Chao Xu, Huan Song, Xiaobo Feng, Liang Ma, Xiaoguang Zhang, Gaocai Li, Congpu Mu, Lei Tan, Zhengdong Zhang, Zhongyuan Liu, Zhiqiang Luo, Cao Yang

**Affiliations:** 1 Department of Orthopaedics, Union Hospital, Tongji Medical College, Huazhong University of Science and Technology, Wuhan, 430074 China; 2 College of Materials Science and Engineering, Wuhan Textile University, Wuhan, 430200 China; 3 Otorhinolaryngology Head and Neck Surgery, Wuhan Fourth Hospital, Wuhan, Hubei 430033 China; 4 College of Life Science and Technology, Huazhong University of Science and Technology, Wuhan, 430022 China; 5 School of Clinical Medicine, Department of Orthopedics, Chengdu Medical College, the First Affiliated Hospital of Chengdu Medical College, Chengdu, 610500 China; 6 Center for High Pressure Science, State Key Laboratory of Metastable Materials Science and Technology, Yanshan University, Qinhuangdao, 066004 China

**Keywords:** Biomimetic scaffold, Spatiotemporal regulation, Innervation, Osteosarcoma, Bone regeneration

## Abstract

The complexity of repairing large segment defects and eradicating residual tumor cell puts the osteosarcoma clinical management challenging. Current biomaterial design often overlooks the crucial role of precisely regulating innervation in bone regeneration. Here, we develop a Germanium Selenium (GeSe) co-doped polylactic acid (PLA) nanofiber membrane-coated tricalcium phosphate bioceramic scaffold (TCP-PLA/GeSe) that mimics the bone-periosteum structure. This biomimetic scaffold offers a dual functionality, combining piezoelectric and photothermal conversion capabilities while remaining biodegradable. When subjected to ultrasound irradiation, the US-electric stimulation of TCP-PLA/GeSe enables spatiotemporal control of neurogenic differentiation. This feature supports early innervation during bone formation, promoting early neurogenic differentiation of Schwann cells (SCs) by increasing intracellular Ca^2+^ and subsequently activating the PI3K-Akt and Ras signaling pathways. The biomimetic scaffold also demonstrates exceptional osteogenic differentiation potential under ultrasound irradiation. In rabbit model of large segment bone defects, the TCP-PLA/GeSe demonstrates promoted osteogenesis and nerve fibre ingrowth. The combined attributes of high photothermal conversion capacity and the sustained release of anti-tumor selenium from the TCP-PLA/GeSe enable the synergistic eradication of osteosarcoma both in vitro and in vivo. This strategy provides new insights on designing advanced biomaterials of repairing large segment bone defect and osteosarcoma.

**Supplementary Information:**

The online version contains supplementary material available at 10.1186/s12951-024-02430-7.

## Introduction

Osteosarcoma (OS) is the most common major malignant bone tumor, with a high metastasis rate, a poor prognosis, a low survival rate, and the highest incidence in children and adolescents, which occurs most frequently near the metaphysis of the femur, tibia, and humerus [1]. The most frequently used treatment for osteosarcoma is the combination of surgical resection and chemotherapy/radiation [2]. Large segment bone defects are common after excision, and some tumor cells may still be present. The presence of residual tumor cells increases the risk of osteosarcoma recurrence [3]. Postoperative radiotherapy and chemo/radiotherapy effectively eliminate tumor cells but often lead to numerous adverse effects, which can impede the process of bone healing [4]. Consequently, it is important to develop more effective and advanced platforms that eradicate residual tumor cells and effectively stimulate bone regeneration.

Bones are thickly innervated by peripheral sensory and motor nerve fibers innervate [5, 6]. A growing body of evidence suggests that early innervation is important in initiating bone formation [7, 8]. Bone regeneration begins with innervation, releasing neurotransmitters and neurotrophic factors that promote osteoblast differentiation, and angiogenesis [9, 10]. The nerve growth factor (NGF)-neurotrophic tyrosine kinase receptor (TrkA) signaling pathways regulate ossification. Knocking down NGF or inhibition of TrkA signaling in osteochondral progenitor cells reduces skeletal nerve ingrowth, vascular regeneration, and bone mass loss in mice [11]. Several animal and molecular studies have shown that NGF-TrkA signaling is important in bone regeneration and remodeling [12]. Bone vascularization and fracture healing are hampered when local nerve repair is suppressed. Due to its attachment to the surface of bone and abundant nerve supply, the periosteum plays a crucial regulatory role in bone regeneration [13]. The current design of biomaterials has neglected the structure and function of the periosteum, biomaterials with bone-periosteum construction can imitate natural bone tissue in structure and function, and have hold great promise for bone regeneration [14]. Unlike patients with common bone defects, osteosarcoma patients have a worse microenvironment for tissue regeneration [15]. Hence, achieving early innervation in individuals who have undergone surgery for osteosarcoma may be a difficult task. Recently, several multifunctional biomaterials have been developed with the goals of preventing bone tumor growth and fostering the process of bone repair. The conventional design approach involves the integration of conventional osteogenic scaffolds with anti-tumor materials. While these materials exhibit commendable anti-tumor properties, their capacity for promoting osteogenesis in the context of extensive bone defects remains a significant challenge, primarily due to the absence of precise mechanisms for regulating nerve growth. Research centered on bone materials that enhance nerve regeneration is still in its early stages. Biological components and cells regulate the function of generating neurogenic differentiation [16–18]. However, this approach has some limitations such as issues related to immune rejection and ethical considerations. Electroactive materials can avoid this problem, and degradable electroactive materials have broad application prospects in promoting bone and nerve tissue repair [19–21]. When subjected to ultrasonic activation, piezoelectric materials can deliberately produce electrical stimulation, a feature that has demonstrated its efficacy in precisely regulating the regeneration of nervous tissues [22–24]. To date, no effective strategy has been proposed to achieve controllable spatiotemporal regulated innervated bone regeneration and antitumor effects. Therefore, an ideal scaffold material for osteosarcoma treatment should have excellent piezoelectric properties, anti-tumor ability and be degradable. Piezoelectric copolymers poly(lactic acid) (PLA) is biodegradable, but its poor piezoelectric properties limit its application in tissue engineering [22]. The piezoelectric properties of the piezoelectric polymer can be improved by doping piezoelectric nanomaterials with high piezoelectric coefficient [25]. Studies have shown that Germanium selenide (GeSe) has excellent piezoelectric properties [26]. The piezoelectric coefficient of organic polymer materials such as PLA can be improved by doping GeSe nanosheets [27]. Moreover, GeSe is regarded as degradable in a physiological environment [28]. Selenium is known to be an anti-cancer element as the final degradation product of germanium selenide nanomaterials. Studies have shown that selenium has numerous properties, including tumor prevention, tumor invasion, and metastasis [29]. Selenium has advantages over conventional chemoradiotherapy drugs in that it can slow tumor growth at the same time preserving healthy tissue functionality [30]. Furthermore, GeSe is postulated to have excellent photothermal capabilities [31]. As such, we speculate that GeSe nanomaterials can achieve thermochemical conversion performance and Se release for thermal ion therapy of osteosarcoma. Biomimetic periosteum composed of PLA and GeSe (PLA/GeSe) can promote nerve regeneration through the piezoelectric effect ,and also achieve anti-tumor effects via thermochemical conversion performance and Se release. Recently, the treatment of osteosarcoma based on light and ultrasound has been the focus of scholars [32]. Significantly, germanium and selenium are essential elements for the human body, and when consumed in appropriate amounts, they pose no harm [33, 34].

Therefore, we envision the piezoelectric bone-periosteum-like biomimetic scaffold made of PLA/GeSe as potential biodegradable multifunctional biomaterial for spatiotemporal regulating innervated osteogenesis and photothermal-ion therapy to eradicate tumour cells.

**Fig. 1:**
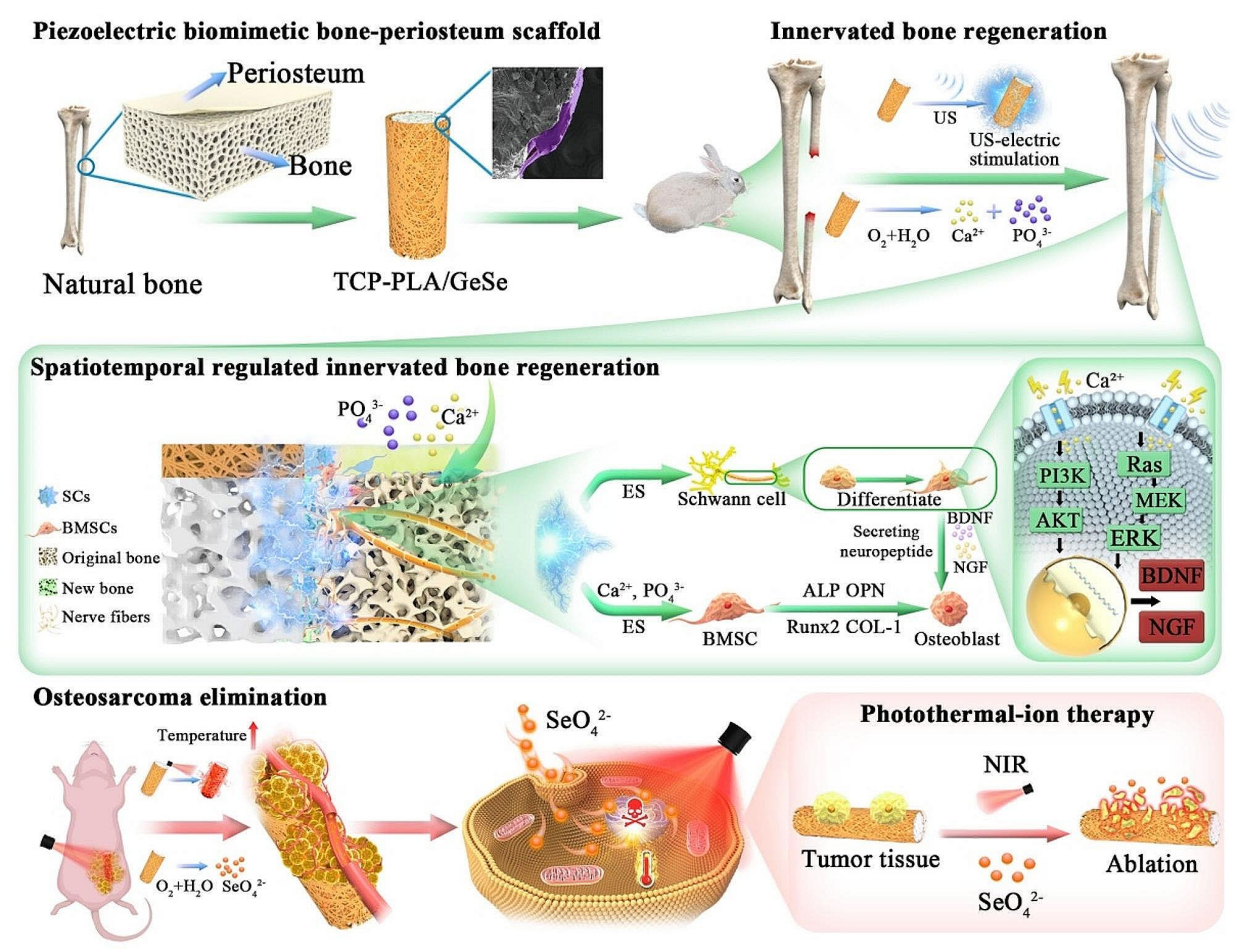
Schematic illustration of the Piezoelectric biomimetic bone-periosteum scaffold (TCP-PLA/GeSe) for spatiotemporal innervated bone regeneration and tumor ablation. Piezoelectric therapy of TCP-PLA/GeSe scaffold with ultrasound (US)-driven would effectively promote early neurogenic differentiation and osteogenesis. The synergistic effect of the piezoelectric effect and TCP bioceramic promotes long-term bone regeneration. Furthermore, the remarkable photothermal-conversion efficiency of the TCP-PLA/GeSe scaffold, coupled with its controlled Se release, enables exceptionally effective tumor eradication. This scaffold, TCP-PLA/GeSe, serves as a captivating biomaterial platform, offering multifunctional capabilities that hold great promise for diverse applications in spatiotemporal innervated bone regeneration and osteosarcoma treatment

Here, electrospinning technology was used to develop piezoelectric bone-periosteum-like biomimetic poly (lactic acid) PLA/GeSe nanofiber films coated porous TCP scaffold (TCP-PLA/GeSe) for reliable temporally regulated innervated bone regeneration and photothermal-ion therapy for Osteosarcoma (Fig. 1). As a conventional osteogenic material, the porous structure of the TCP bioceramic and continuous release of calcium and phosphorus can provide long-term support for bone regeneration [35]. Piezoelectric therapy of PLA/GeSe nanofiber membrane driven by ultrasound (US) can improve early neurogenic differentiation and osteogenic differentiation. The combined action of the piezoelectric effect and TCP bioceramic fosters sustained bone regeneration over the long term. On the other hand, the effective tumor-killing mechanism of photothermal-ion therapy relies on the impressive photothermal-conversion rate of PLA/GeSe nanofiber membrane and the subsequent release of Se. The unique properties of TCP-PLA/GeSe scaffolds have been comprehensively evaluated and substantiated (in vitro and in vivo) for their improved anticancer effects and spatiotemporal regulated innervated bone regeneration.

## Result and discussion

### Fabrication and characterizations of TCP-PLA/GeSe scaffolds

**Fig. 2:**
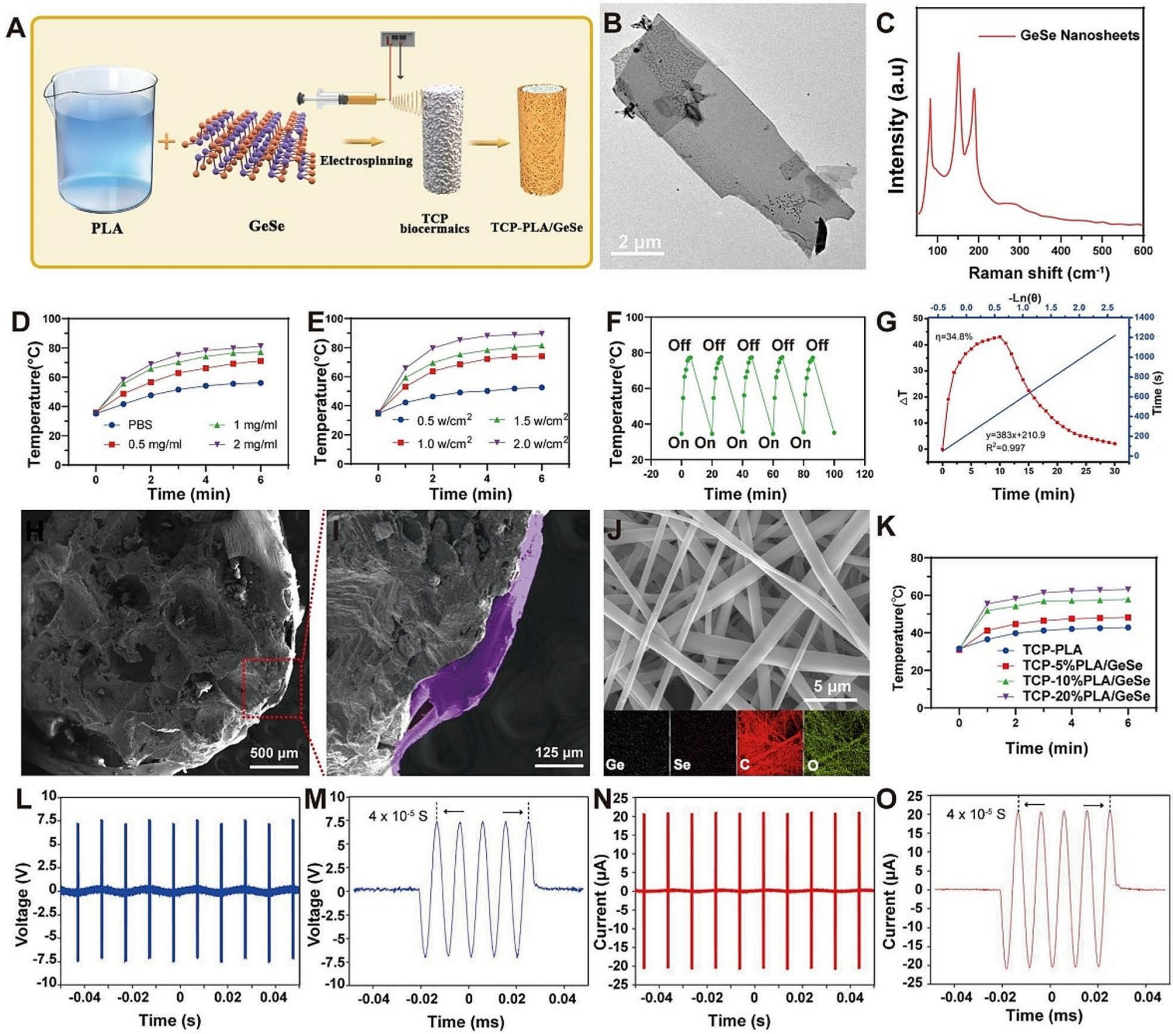
Characterizations of GeSe nanosheets and TCP-PLA/GeSe scaffold. (**A**) Schematic illustration of the fabrication of TCP-PLA/GeSe scaffolds. (**B**) TEM images of GeSe nanosheets. (**C**) The Raman spectroscopy of GeSe nanosheets. (**D**) Temperature changing curves of GeSe nanosheets in vitro at different concentrations. (**E**) Temperature changing curves of GeSe nanosheets in vitro at different laser power densities of NIR. (**F**) Temperature profiles of scaffolds during four lasers on/off cycles. (**G**) Heating and cooling curve of the aqueous dispersions of GeSe nanosheets (1.0 mg/mL) under 808 nm (1.0 W/cm^2^) laser irradiation, and the linear fitting curve of time and -Ln (ɵ) acquired from the cooling period. (**H**-**I**) The SEM images of TCP-10%PLA/GeSe scaffold. (**J**) SEM image and element mapping of PLA/10%GeSe nanofiber membrane. (**K**) Temperature changing curves of TCP-PLA/GeSe scaffolds in vitro at different concentrations (**L**-**M**) The open-circuit voltage generated by TCP-10%PLA/GeSe scaffold under US excitation. (**N**-**O**) The short-circuit current generated by TCP-10%PLA/GeSe scaffold under US excitation

The creation process of TCP-PLA/GeSe scaffolds, as illustrated in Fig. 2A, involves a concise method where GeSe nanosheets are blended with a PLA solution, and these resultant solutions are subsequently employed in the electrospinning process. The PLA/GeSe nanofiber membrane-coated TCP bioceramics (TCP-PLA/GeSe) was further fabricated by electrospinning to prepare a biomimetic structure resembling bone and periosteum. Calcium phosphate bioceramics, such as TCP bioceramics, as a three-dimensional scaffold material,is a common bone biomaterial, which can provide a good supporting structure for bone cell growth and new bone formation [36]. The degradation products are phosphate and calcium ions of calcium phosphate bioceramics which can further promote the formation of new bones [20]. By introducing various anti-tumor ingredients, the role of Calcium phosphate materials in the treatment of osteosarcoma has been widely explored [37]. The piezoelectric effect of PLA/GeSe nanofiber membrane can promote nerve and bone regeneration, and has biological functions similar to periosteum, so it is used to simulate the structure of periosteum. What’s more, PLA/GeSe nanofiber membrane PLA/GeSe nanofiber membrane also has anti-tumor potential. Therefore, biomimetic bone-periosteum scaffold composed of TCP ceramics and PLA/GeSe nanofiber membrane will provide a new strategy for the treatment of osteosarcoma. GeSe nanosheets have typical lateral dimensions of approximately 5 μm and a two-dimensional sheet morphology, from and transmission electron microscopy (TEM) (Fig. 2B) images and scanning electron microscope (SEM) images (Figure S1A, Supporting Information). X-ray diffraction (XRD) was used to evaluate the crystal structure of GeSe nanosheets. The GeSe diffraction peaks (Figure S1B, Supporting Information) conform to the standard pattern (JCPDS no. 48-1226), confirming the high purity of the initial sample and supporting previous research [38]. Raman spectroscopy was used to further characterize GeSe nanosheets. Figure 2C shows a typical Raman spectrum. The distinct peaks at 83 and 189 cm^− 1^ were assigned to the A_g_^1^ and A_g_^2^ modes, whereas the peak at 152 cm^− 1^ was assigned to the B_3_^g^ mode, which is consistent with previous research [39].

The photothermal conversion capacity of GeSe nanosheets was then tested. GeSe nanosheets were added to PBS at different initial integration concentrations (0.5, 1.0, 1.5, and 2.0 mg/mL), before exposing to an 808 nm laser irradiation (6 min, 1.0 W/cm^2^). The final temperature increased to 74.1 °C at the low concentration of GeSe solution (0.5 mg/mL) (Fig. 2D), whereas no significant change was noted in PBS. This shows the effective and rapid photothermal conversion capacity of GeSe nanosheets. The photothermal effect of the GeSe nanosheets was also dependent on the power density of laser irradiation (Fig. 2E). The final temperature of the GeSe solution (1 mg/mL) increased quickly from 35 to 81 °C at varied power densities of NIR irradiation from 0.5 to 2.0 W/cm^2^ (808 nm, 6 min). To further investigate photothermal stability (Fig. 2F), the photothermal capability of GeSe solution (1 mg/mL) underwent five irradiation cycles and demonstrated negligible variation, indicating favorable photothermal stability. Further, we assessed the response of temperature change (ΔT) to the NIR laser within 10 min and obtained linear time data -ln(ɵ) from the cooling period study (Fig. 2G). GeSe nanosheets had an excellent photothermal conversion efficiency of 34.8%, exceeding that of conventional photothermal conversion materials including Pt NPs, and Au nanorods [40, 41].

Subsequently, GeSe nanosheets were dissolved in PLA solution to constitute PLA/GeSe solution mixtures. The obtained composite solutions with different mass percentages of GeSe in PLA (5%, 10%, 20%) were used for electrospinning. The PLA/GeSe nanofiber membrane-coated TCP bioceramics (TCP-PLA/GeSe) was further fabricated by electrospinning to form a biomimetic structure resembling bone and periosteum (Figure S2A, Supporting Information, TCP-10%PLA/GeSe**)**. A scanning electron microscope (SEM) was used to analyze the pore morphology of the TCP-10%PLA/GeSe scaffold (Fig. 2H). TCP bioceramic had a high pore structure, pore size exceeding 100 μm, which promoted cell growth and nutrients as well as oxygen exchange [42]. A double-layer structure made up of the TCP bioceramic scaffold and 10%PLA/GeSe membrane could be observed (Fig. 2I), the PLA/GeSe membrane has a purple marking. The SEM and element mapping was also used to assess the structure and composition of nanofiber membranes. The result showed that the structure of nanofiber membranes comprised fibers oriented in random directions, without aggregation or nodes, these fibers present a relatively smooth interconnected surface, and the elements carbon (C), oxygen (O), germanium (Ge), and selenium (Se) are distributed evenly across the membrane (Fig. 2J). Additionally, we conducted SEM analysis on nanofiber membrane coatings with varying Ge concentrations. The outcomes revealed that the structural stability remained consistent across different Ge concentrations, indicating that Ge concentration did not have a significant impact on the membrane structures (Figure S2B-D, Supporting Information).

Notably, GeSe have previously demonstrated high photothermal conversion efficiency in experiments. This suggests that TCP-PLA/GeSe scaffolds can efficiently convert light energy into heat, thereby enabling photothermal therapy to effectively ablate tumors. Under near-infrared (NIR) irradiation, TCP-PLA/GeSe scaffold temperature increased from 32.1 ℃ to 63.1 ℃ with the 808 nm laser for 6 min (TCP-20% PLA/GeSe, power density = 1.0 W/cm^2^, Fig. 2K). In contrast, the TCP-PLA scaffold exhibited minimal photothermal effects, with a mere approximate 10 ℃ temperature rise observed at a comparable power density. This observation further underscores the crucial role played by GeSe nanosheets in photothermal treatment. Besides the PLA/GeSe ratio, the photothermal properties of composite scaffold also depend on the laser power density. The higher the laser power, the higher the temperature increase of TCP-PLA/GeSe (Figure S3A, Supporting Information). The photothermal stability of the TCP-10%PLA/GeSe scaffold was also confirmed through five lasers on/off cycles (Figure S3B, Supporting Information**)**. Studies have demonstrated that tumor cells could be killed at around 45 ℃ [43]; TCP-10% PLA/GeSe increased to 51.8 ℃ with the 808 nm laser just in 1 min. Therefore, TCP-10%PLA/GeSe has good photothermal stability and promotes continuous photothermal ablation of bone tumors. BMSCs and Schwann cells were used to evaluate the cytocompatibility of TCP-PLA/GeSe with different GeSe concentrations. As shown in Figure S4A, B, Supporting Information, both BMSCs and SCs were effectively proliferated on different scaffolds, whereas TCP-10% PLA/GeSe demonstrated the best proliferation, unlike other groups. Noteworthy, TCP-20% PLA/GeSe had significant cytotoxicity. Therefore, TCP-10% PLA/GeSe was selected for subsequent testing.

The electrical output capacity of TCP-PLA/GeSe scaffold was evaluated in deionized water, and measured under ultrasonic excitation, with the ultrasound (US) parameter chosen for the study being a frequency of 100 MHz, pulse width of 50 µs, pulse interval of 10 ms and intensity of 0.7 W/cm². Meanwhile, the open circuit voltage and short circuit current of the TCP-10%PLA/GeSe scaffold were recorded. As shown in Fig. 2L and N, the output voltage and short circuit current of the TCP-PLA/GeSe scaffold reached 7.5 V and 22 µA, respectively. Furthermore, the output voltage and current generated from the piezoelectric scaffold were both in sinusoidal waves with signa intervals of about 10^− 5^ s, suggesting their real-time response to ultrasound vibration (Fig. 2M and O). As shown in Figure S5A, B, Supporting Information, the output voltage and short circuit current of the TCP-PLA scaffold reached 3.3 V and 2.2 µA, respectively. Therefore, incorporation of GeSe into PLA nanofibers can significantly enhance piezoelectric properties. The ion release from biodegradable TCP-PLA/GeSe scaffold was investigated using an inductively coupled plasma-atomic emission spectrometer (ICP-OES). Normal physiological microenvironment is neutral, but tumors usually lead to acidic microenvironment, which can affect the degradation of nanomaterials [44]. Therefore, we measured the ion release of the TCP-PLA/GeSe scaffold at PH 7.4 and 6.5. The cumulative profiles demonstrated that the Ca, P Ge, and Se ions could be gradually released from the TCP-PLA/GeSe scaffold at PH 7.4 and 6.5, In acidic environment, the release of TCP-PLA/GeSe scaffold ions increased, which was consistent with the report ( Figure S5C,D, Supporting Information) [45].

### The spatiotemporal regulated neurogenic differentiation of SCs on TCP-PLA/GeSe scaffolds under US excitation

Schwann cells (SCs) perform key functions in neural regeneration through myelination and secretion of many neurotrophic chemicals [46]. Precisely controlling the early stages of nerve regeneration is essential for triggering bone regeneration and facilitating the healing of significant bone defects. Nerve regeneration can be effectively improved by in vivo electrical stimulation, which can be generated by driving piezoelectric materials with ultrasound to generate piezoelectric effects [47]. Therefore, we stimulated the TCP-PLA/GeSe scaffold with ultrasound to create electrical stimulation and achieve an spatiotemporal regulation of neurogenic differentiation. Considering the role of innervation in bone regeneration, SCs were seeded on TCP-PLA and TCP-PLA/GeSe scaffolds irradiated with/without ultrasound to assess neurite development capacity. Herein, the ultrasound (US) parameter selected was a frequency of 100 MHz, pulse width of 50 µs, pulse interval of 10 ms, and intensity of 0.7 W/cm². For biocompatibility testing, SCs were seeded on scaffolds, and on the third day, live/dead staining experiments were conducted (Figure S6A, Supporting Information). Live/dead staining results were consistent with that of CCK-8, and SCs seeded in each group had similar viability (Figure S6B, Supporting Information). The findings show that TCP-PLA and TCP-PLA/GeSe scaffolds are biocompatible, and that ultrasonic has negligible negative effects on cell survival.

**Fig. 3:**
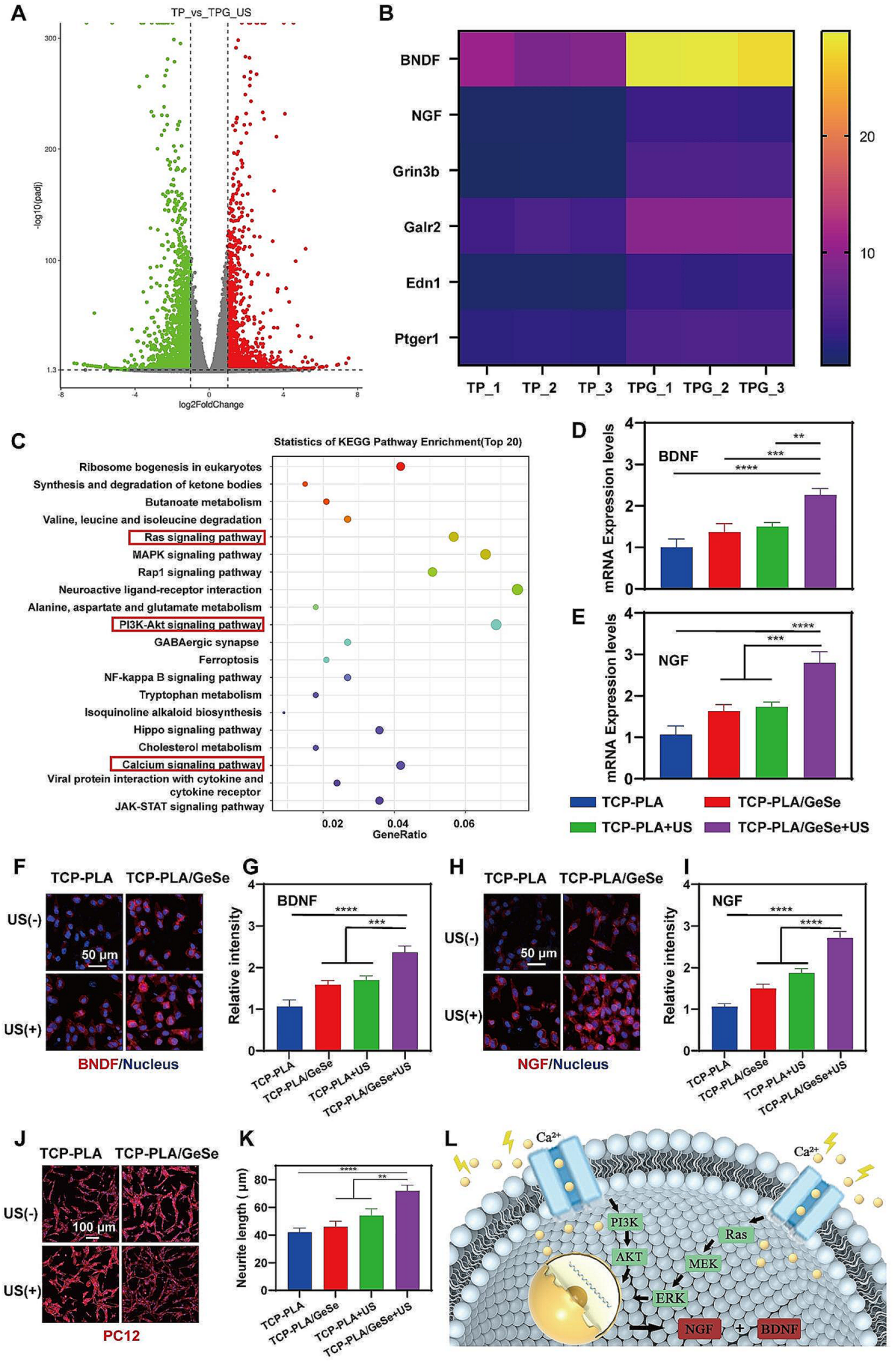
The neurogenic activity of SCs on TCP-PLA/GeSe scaffolds under US excitation. (**A**) The differentially expressed genes(GEGs) between TCP-PLA and TCP-PLA/GeSe + US group. (**B**) The heat map of gene expression for the neurogenic activity between the TCP-PLA and TCP-PLA/GeSe + US groups.(**C**) Top 20 KEGG pathways of genes enriched in the TCP-PLA/GeSe + US group. RT-PCR analysis of (**D**) BDNF and (**E**) NGF gene expression in different groups. (**F**) Representative images of BDNF proteins expression of SCs cultured in different groups. (**G**) Statistical analysis of BDNF expression of SCs cultured in different groups. (**H**) Representative images showing NGF proteins expression in SCs cultured under different conditions (**I**) Statistical analysis of NGF expression of SCs cultured in different groups. (**J**) Immunostaining images of PC12 cells cultured in SC-conditioned medium of different groups. (**K**) Statistical analysis of neurite length. (**L**) Schematic illustration of the mechanism of TCP-PLA/GeSe scaffold promoting neurogenic differentiation. *n* = 3. (* *p* < 0.05, ** *p* < 0.01, *** *p* < 0.001)

To detect changes in their gene profiles and neural differentiation function, we performed mRNA sequencing (RNA-seq) on Schwann cells co-cultured with TCP-PLA and TCP-PLA/GeSe + US. As shown in Fig. 3A, the expression profiles of the TCP-PLA/GeSe + US group were substantially different from the profiles of the TCP-PLA group. Unlike the TCP-PLA group, there were 1151 downregulated and 1721 upregulated differentially-expressed genes (GEGs), indicating that SCs treated with TCP-PLA/GeSe under US excitation influence expression genetic cell profiles. Figure 3B shows the heat map of the gene expression for the neurogenic between the TCP-PLA and TCP-PLA/GeSe + US groups. In summary, we show that SCs on TCP-PLA/GeSe scaffolds under US excitation can alter the expression of gene profiles and up-regulate the function associated with nerve differentiation. Using the Kyoto Encyclopedia of Genes and Genomes (KEGG), we discovered that after TCP-PLA/GeSe + US treatment, there was prominent gene enrichment in the PI3K-Akt, Ras-involved pathways and calcium signaling pathways (Fig. 3C). PI3K-Akt and Ras are neurotrophic signaling pathways, closely related to nerve differentiation, axon growth, dendrite branching, and synaptic formation [48, 49]. One of the primary mechanisms of electrical stimulation in nerve differentiation is the involvement of the calcium signaling pathway [50]. Subsequently, RT-qPCR was performed to determine gene expression in SCs associated with neurogenesis (BDNF and NGF). During SC myelination, BDNF and NGF expression becomes upregulated, resulting in increased axon development and peripheral nerve regeneration [51]. Figure 3D-E shows that TCP-PLA/GeSe + US significantly up-regulating the expression of BDNF and NGF genes. In further tests, we quantified the protein expression on SC was evaluated by immunofluorescence staining. SCs in the TCP-PLA/GeSe + US group significantly expressed more neurogenic-associated proteins (BDNF and NGF) than that in the TCP-PLA + US group (Fig. 3F-I).

The effect of TCP-PLA/GeSe scaffolds irradiated with ultrasound on neurite outgrowth was examined in PC12 cells cultured in an SC-conditioned medium. Each group of PC12 cells showed neurite outgrowth; however, the TCP-PLA/GeSe + US group showed the longest axons (Fig. 3J-K). This may be explained by the higher concentration of neural factors in the Schwann cell-conditioned medium of the TCP-PLA/GeSe + US group, which effectively promotes nerve regeneration. Besides regulating nerve regeneration, SCs play a direct role in osteogenic regeneration [52]. The paracrine activity of SCs can affect osteogenic development in mouse bone stem cells, releasing a range of substances, including BDNF, NGF etc., which are involved in the regulation of osteogenesis [53, 54]. Therefore, we used SC medium conditioned on different groups to detect osteogenic differentiation of BMSCs. The results of ALP and ARS (Figure S7, Supporting Information) staining revealed that SC medium conditioned on TCP-PLA/GeSe + US group promoted osteogenic differentiation of BMSC. Overall, SCs in TCP-PLA/GeSe + US groups had the highest neurogenic activity under US excitation, and TCP-PLA/GeSe scaffold may target PI3K/AKT and Ras pathways by increasing intracellular Ca^2+^ concentration, thereby promoting neurogenic differentiation (Fig. 3L).

### Osteogenic activity of BMSCs on TCP-PLA/GeSe scaffolds under US excitation

**Fig. 4:**
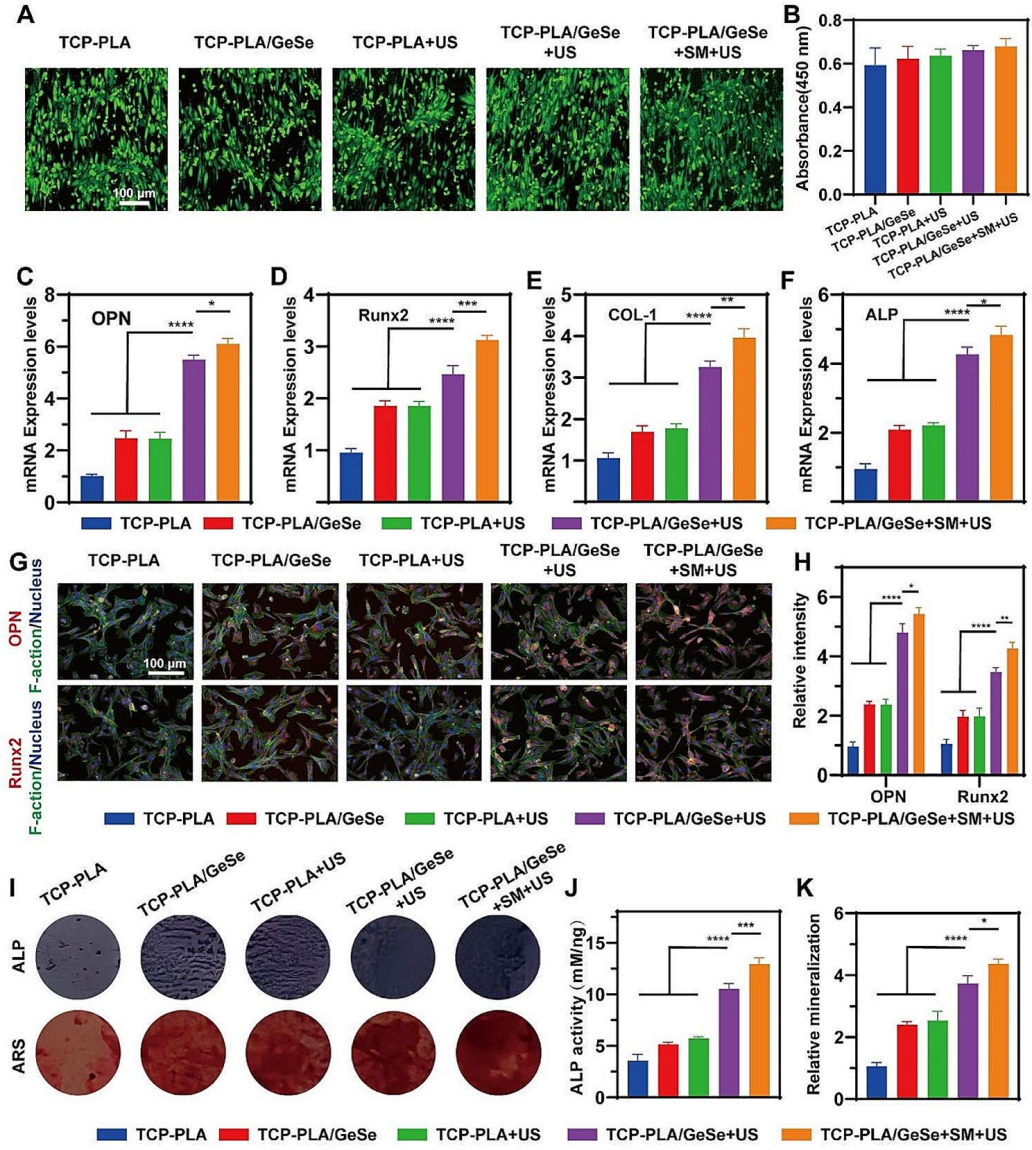
Osteogenic activity of BMSCs on TCP-PLA/GeSe scaffolds under US excitation. (**A**) Fluorescent images of BMSCs on TCP-PLA and TCP-PLA/GeSe scaffolds with/without US excitation. (**B**) Viability of BMSCs on TCP-PLA and TCP-PLA/GeSe scaffolds with/without US irritation. RT-PCR analysis of (**C**) OPN, (**D**) Runx2, (**E**) COL-1, and (**F+**) ALP gene expression in different groups. (**G**) Typical fluorescence pictures depicting OPN and Runx2 protein expression in different groups. (**H**) The statistical analysis of OPN and Runx2 expression in different groups. (**I**) ALP and ARS staining of BMSCs in different groups. (**J**) ALP and (**K**) ARS quantitative assay in different groups. *n* = 3. (* *p* < 0.05, ** *p* < 0.01, *** *p* < 0.001)

To further evaluate the biocompatibility of the TCP-PLA/GeSe scaffold under US excitation, BMSCs were planted on it. On day 3 after seeding, live/dead staining revealed little dead cells on the scaffold (Fig. 4A). TCP-PLA/GeSe + SM + US refers to PC12 cells cultured under SC-conditioned medium. The viability of BMSCs of the different groups was evaluated using CCK-8 assays to assess cell activity (Fig. 4B). Moreover, there was no significant difference in cell viability between the groups. The results demonstrate the biocompatibility of TCP-PLA/GeSe scaffolds and the negligible negative effects of ultrasonic on cell survival.

The capacity of biomaterials to promote osteogenic differentiation is important for accelerating bone defect repair. First, BMSCs were seeded onto the samples for 14 days, and the effects of TCP-PLA/GeSe scaffolds on osteogenic gene expression were investigated using RT-PCR (ALP, COL-I, Runx-2, OPN). Compared with other three groups, the TCP-PLA/GeSe + US group and TCP-PLA/GeSe + SM + US group had considerably increased osteogenesis gene expression, and the highest expression was recorded in the TCP-PLA/GeSe + SM + US group. (Fig. 4C-F). This illustrates that when subjected to ultrasonic stimulation, the scaffold can augment osteogenic differentiation, and the neurotransmitters released during Schwann cell differentiation positively influence this osteogenic differentiation process. Runx-2 and OPN expression in BMSCs were further examined using immunofluorescence labeling. The TCP-PLA/GeSe + SM + US group had the highest levels of OPN and Runx-2 expression (Fig. 4G-H). Furthermore, ALP staining and calcium nodule deposition staining were used to test the osteogenic differentiation of BMSCs on scaffolds (Fig. 4I). The ALP and ARS statistical results were comparable with the immunofluorescence results (Fig. 4J-K). Conclusively, these results indicate that TCP-PLA/GeSe scaffolds irradiated with ultrasound harbor excellent osteogenic activity in vitro, and neurogenic differentiation contributes to osteoblast differentiation.

### Performance of repairing large segmental bone defects of the TCP-PLA/GeSe scaffolds under US excitation

**Fig. 5:**
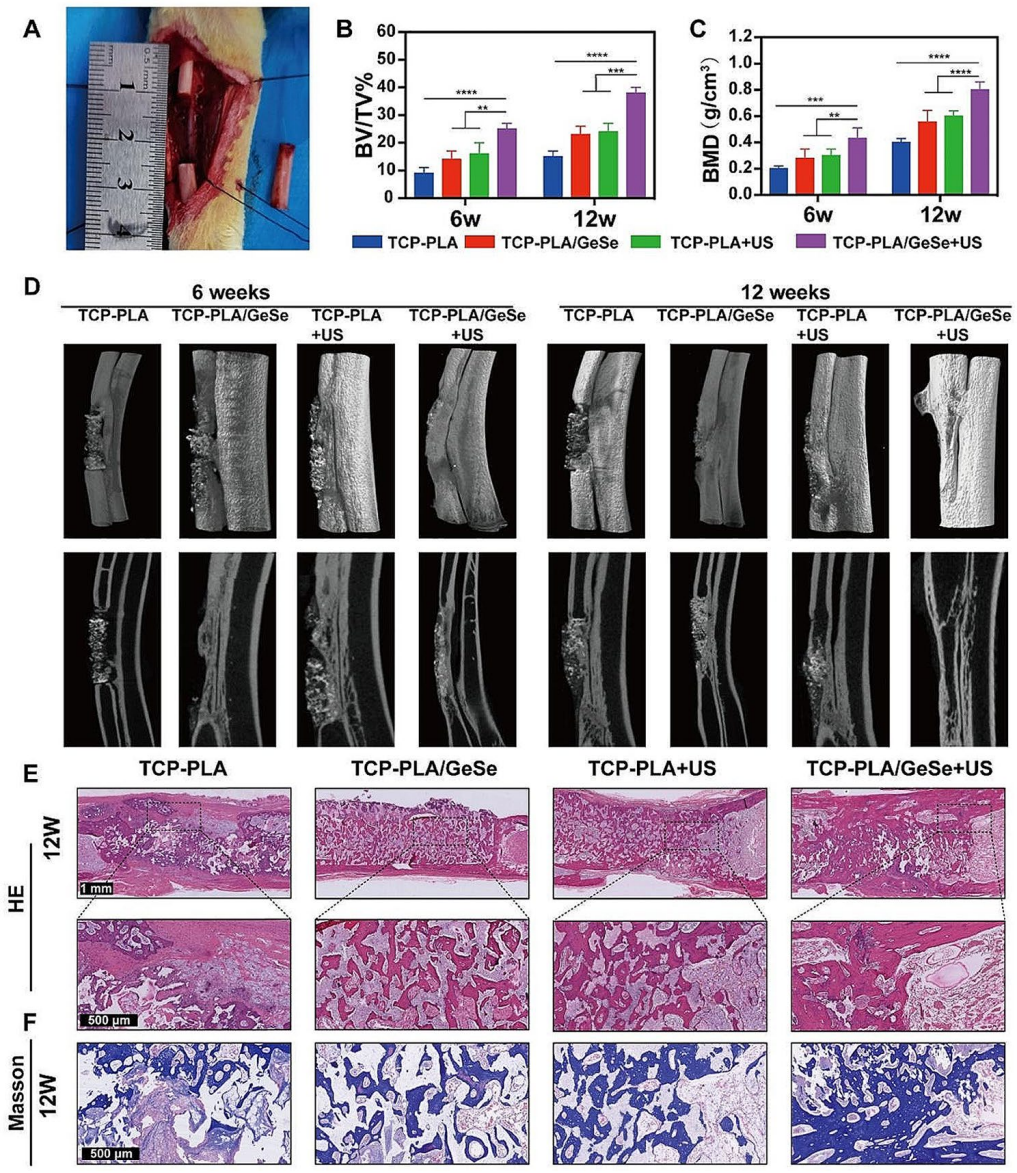
Performance of repairing large segmental bone defects of TCP-PLA/GeSe scaffolds under US excitation. (**A**) Large segmental bone defect in the middle radius of New Zealand rabbits. (**B**, **C**) Micro-CT quantification of newly formed bone. (**D**) 3D-reconstructed images of newly formed bone in different groups. (**E**) The regeneration of bone was evaluated 12 weeks after surgery using H&E and (**F**) Masson stains. *n* = 3. (* *p* < 0.05, ** *p* < 0.01, *** *p* < 0.001, **** *p* < 0.0001)

**Fig. 6:**
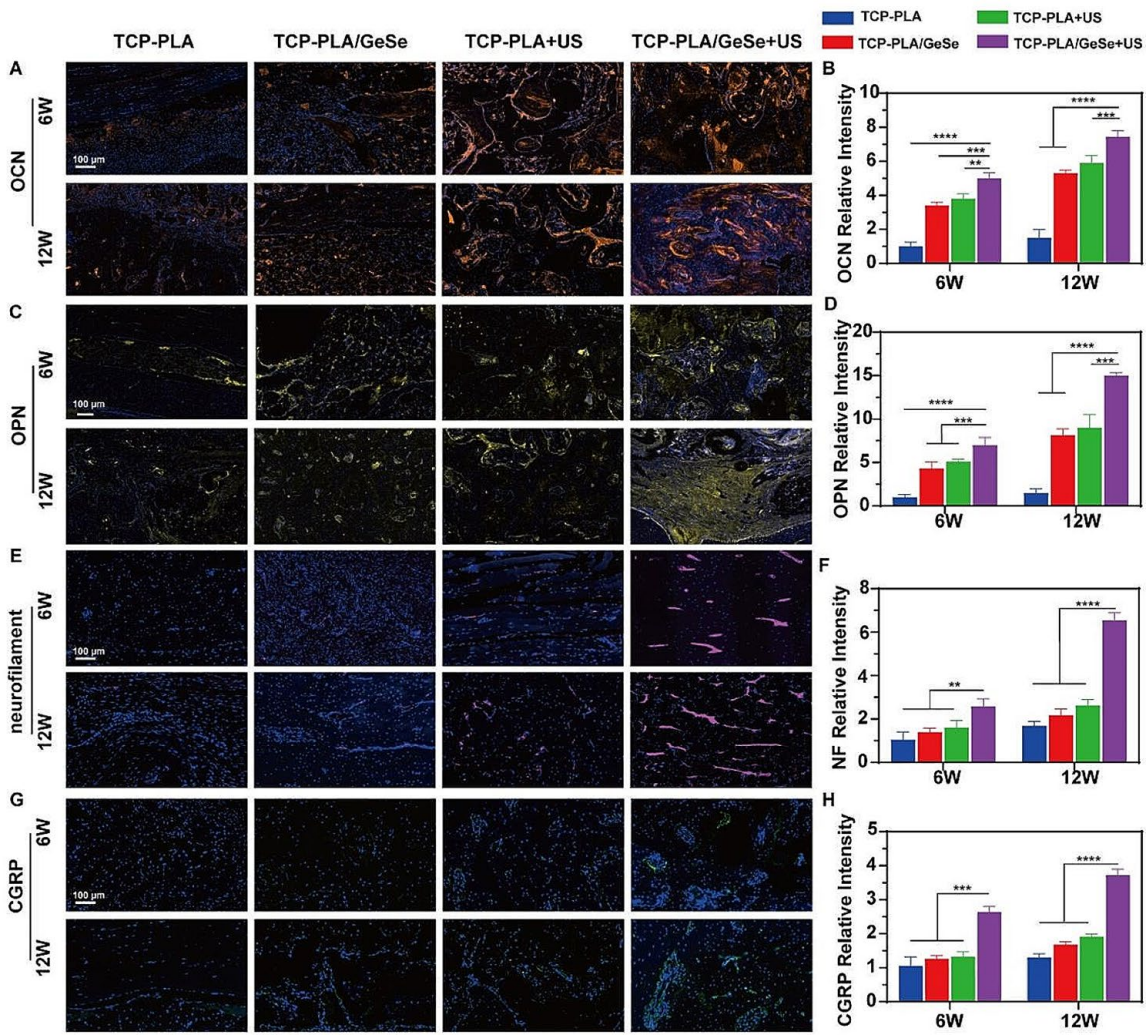
Immunohistological analysis of osteogenic and neurogenic activities. (**A**) OCN and (**C**) OPN staining of bone defect at 6 weeks and 12 weeks. Quantitative statistical analysis of the (**B**) OCN and (**D**) OPN at 6 and 12 weeks in different groups. (**E**) Neurofilament and (**G**) CGRP staining of bone defect at 6 weeks and 12 weeks. Quantitative statistical analysis of the (**F**) Neurofilament and (**H**) CGRP at 6 and 12 weeks in different groups. *n* = 3. (* *p* < 0.05, ** *p* < 0.01, *** *p* < 0.001, **** *p* < 0.0001)

Osteosarcoma surgery often results in a large segment bone defect. We, therefore, prepared a large segment bone defect model in the middle radius of New Zealand rabbits to investigate the bone repair capacity of TCP-PLA/GeSe scaffolds under US excitation. A large segmental bone defect in the mid-radius of New Zealand rabbits was developed to assess bone regeneration capacity. In the middle radius of New Zealand rabbits, a segmental bone defect with a length of 15 mm was produced (Fig. 5A), after which the scaffolds were implanted. At 6 and 12 weeks after scaffold implantation, micro-CT was used to assess newly generated bone formation in different groups (Fig. 5D). All four groups showed ingrowth of newly formed bone after 6 weeks. The TCP-PLA/GeSe + US group, however, demonstrated the ability of the newly formed bone to connect the upper and lower parts of the radius surrounding the defect. The formation of a cavity-like structure was substantial in the TCP-PLA/GeSe + US group. However, it’s crucial to note that the newly formed bone exhibits a markedly distinct morphology compared to native bone tissue. After 12 weeks, all groups showed an increase in new bone tissue unlike after 6 weeks. Although the defect region was filled with new bone in the TCP-PLA, TCP-PLA/GeSe, and TCP-PLA + US groups, the newly formed bone in the TCP-PLA/GeSe + US group completely connected the upper and lower portions of the radius surrounding the defect. Additionally, a medullary cavity was visible in this group. In each group, both the bone score value (BV/TV%) and bone mineral density (BMD) increased over time. Specifically, BV/TV% and BMD were significantly higher in the TCP-PLA/GeSe + US groups than other groups at both 6 and 12 weeks (Fig. 5B-C). At 6 and 12 weeks, statistical analysis of newly formed bone of the TCP-PLA/GeSe + US group was statistically higher than that of the TCP-PLA + US and PLA/GeSe groups. However, no significant difference in the BV/TV% and BMD was noted between TCP-PLA + US and TCP-PLA/GeSe groups at 6 and 12 weeks. The results from HE and Masson staining (Fig. 5E) and (Figure S8, Supporting Information) were also similar to quantitative micro-CT data. The TCP-PLA/GeSe + US group had substantial repair of large segmental bone defects, as revealed by histological analysis. Osteogenic markers including, OCN (osteocalcin) and OPN (osteopontin) were analyzed to estimate the osteogenic capacity of each group. The TCP-PLA/GeSe + US group had the greatest OCN (osteocalcin) expression, as shown by the orange color in Fig. 6A-B. Figure 6C and D show similar outcomes for OPN (osteopontin) expression, denoted by the yellow. At 6 and 12 weeks, both the TCP-PLA/GeSe scaffold and ultrasound stimulated bone regeneration. Notably, the TCP-PLA/GeSe scaffold and ultrasound combination produced a larger volume of regenerated bone. This information suggests that the TCP-PLA/GeSe scaffold and ultrasound can interact with one another, stimulating new bone formation. These results are consistent with in vitro research and provide additional support for the observed effects.

Nerve fibers play an important role in bone reconstruction. Neurofilament is an important cytoskeletal component of neurons and axons commonly used to determine nerve fiber regeneration [55]. Immunofluorescence staining was conducted to evaluate neurofilament ingrowth during bone regeneration. As shown in Fig. 6E, the most numerous nerve fibers could be observed in bone tissue of the TCP-PLA/GeSe + US group (red) at 6 and 12 weeks. Moreover, the quantitative analysis further confirmed that TCP-PLA/GeSe + US significantly induced nerve fibers into the bone defect area, resulting in increased innervation (Fig. 6F). We further defined the type of regenerated nerve fibers. Previous studies have shown that the primary nerve fibers penetrating a repairing bone defect include CGRP+ (calcitonin gene-related peptide) peptidergic nociceptors, which are necessary for bone regeneration [56, 57]. Figure 6G shows the presence of CGRP-positive nerve fibers within the bone defect area of the TCP-PLA/GeSe + US group. Moreover, quantitative statistical analysis in Fig. 6H indicates that the TCP-PLA/GeSe + US group has the highest area of positive CGRP at both 6 and 12 weeks. The presence of primarily CGRP-positive nerve fibers in the regenerated tissue reflects their vital role in promoting bone repair and maintaining bone homeostasis. CGRP-positive nerve fibers have been identified for their involvement in modulating inflammation, angiogenesis, and osteogenesis, which are key processes for successful bone regeneration. Their presence suggests that the TCP-PLA/GeSe scaffold under US excitation can promote the growth and infiltration of nerve fibers, and TCP-PLA/GeSe scaffold contribute to the regenerative process, ultimately enhancing bone defect repair by spatiotemporal regulation of early innervation. In summary, the TCP-PLA/GeSe scaffold can spatiotemporal regulation the ingrowth of CGRP-positive nerve fibers and promote the repair of large-segment bone defects. This feature is specifically beneficial for bone defect treatment and repair after osteosarcoma surgery. By promoting the ingrowth of CGRP-positive nerve fibers, the TCP-PLA/GeSe scaffold promotes the regenerative process, providing promising prospects for successful large segmental bone defect repair in patients undergoing osteosarcoma surgery.

### In vitro and in vivo tumor therapeutic effect of TCP-PLA/GeSe scaffolds

**Fig. 7:**
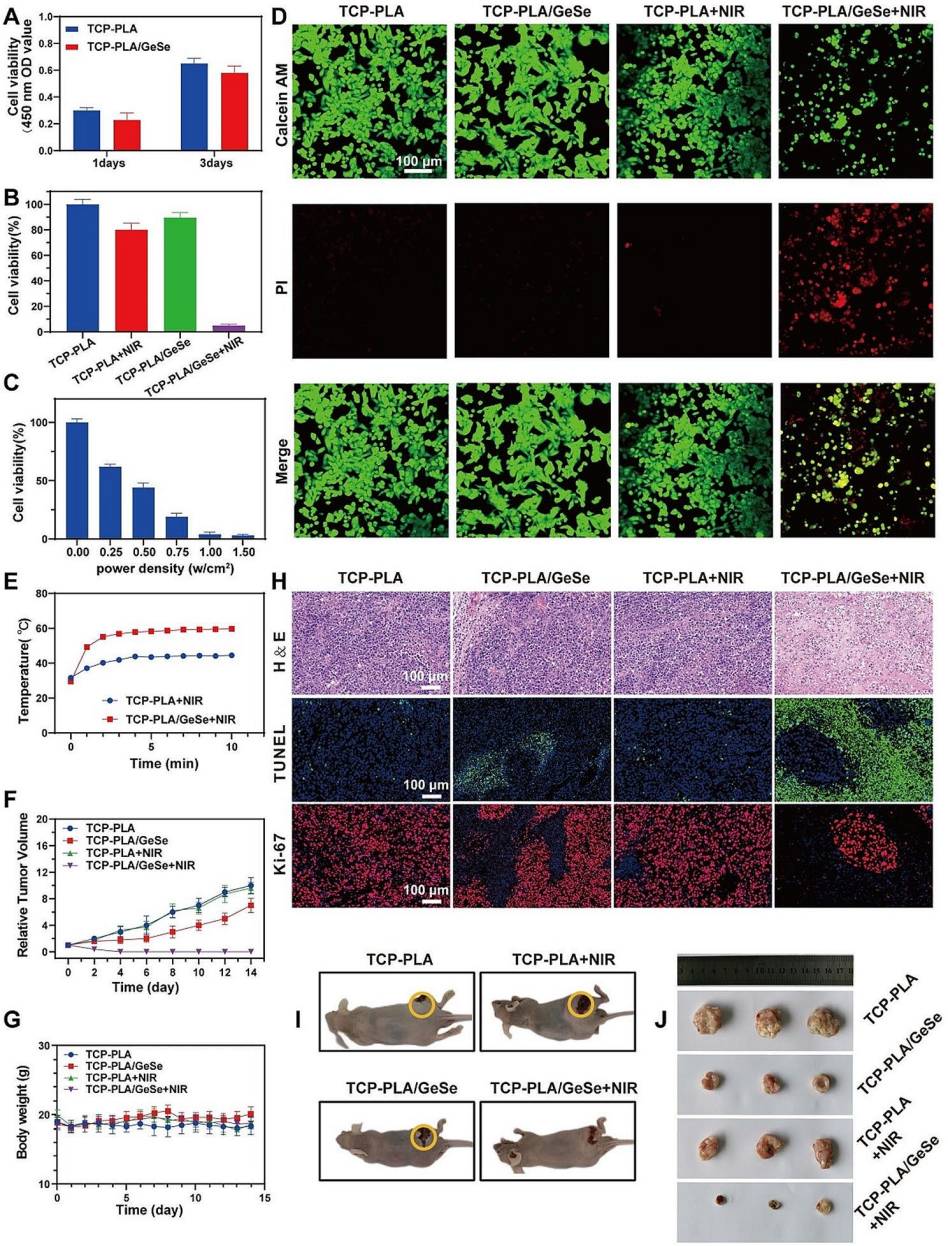
In vitro and in vivo anti-tumor effects of TCP-PLA/GeSe scaffolds. (**A**) CCK-8 of Saos-2 adhered to the TCP-PLA/GeSe scaffold for proliferation within 3 day. (**B**) CCK-8 of Saos-2 treated with different group. (**C**) Viability assay of Saos-2 cells exposed to different power densities of NIR for 6 min. (**D**) Confocal image of dead-viable staining of cells on TCP-PLA/GeSe scaffold. (**E**) Temperature changing curves of mice subjected to different treatments. (**F**) Relative tumor-growth curves of mice across various groups following different treatments. (**G**) Changes in body weight of mice within different groups. (**H**) H&E, TUNEL (apoptosis) and Ki-67 staining were performed in tumor tissues of mice in each groups. Photographs of (**I**) Tumor-bearing mice, (**J**) Resected tumors from different groups at day 14. *n* = 3

In vitro, scaffold cytotoxicity and ability to eradicate tumor cells were measured using the CCK-8 test. On a TCP-PLA/GeSe scaffold, Saos-2 cells were grown before exposure to NIR laser irradiation. Saos-2 adhered to the TCP-PLA/GeSe scaffold for proliferation within 3 days, increasing significantly compared to day 1 (Fig. 7A). This indicates satisfactory biocompatibility of the TCP-PLA/GeSe scaffold. Moreover, fewer Saos-2 cells were on the TCP-PLA/GeSe scaffold than that on the TCP-PLA scaffold, indicating that the GeSe component may inhibit the survival of Saos-2 cells. The TCP-PLA/GeSe scaffold may release selenium components. Our previous studies showed that selenium can stimulate cells to produce -OH (hydroxyl radicals), promote cell death, and synergistic tumor treatment [58]. These findings suggest that selenium release from the TCP-PLA/GeSe scaffold significantly affects cellular responses, including apoptosis activation and potential treatment uses when combined with other treatment techniques. To further validate the anticancer efficacy of the TCP-PLA/GeSe scaffold, Saos-2 cells were incubated on TCP-PLA and TCP-PLA/GeSe scaffold for 24 h before subsequent irradiation with NIR laser (1.0 W/cm^2^) for 6 min. The TCP-PLA/GeSe scaffold with NIR laser excitation caused more than 90% ablation of Saos-2 cells (Fig. 7B**)**. Figure 7C and Figure S9A, Supporting Information, show the effect evaluation of laser power density and irradiation period on cell viability to completely evaluate the therapeutic prospects of the TCP-PLA/GeSe scaffold. After laser irradiation, fluorescent staining was performed to assess the survival of Saos-2 cells on the TCP-PLA/GeSe scaffold. Propidium iodide (PI; red) and calcein-AM (green) were used to specifically stain dead and alive cells in scaffolds; the TCP-PLA/GeSe scaffold + laser group displayed remarkable red fluorescence (Fig. 7D), indicating severely damaged Saos-2 cells by TCP-PLA/GeSe scaffold under laser irradiation. These results strongly indicate that TCP-PLA/GeSe scaffold can profoundly eliminate tumor cells in vitro when exposed to NIR laser.

The therapeutic potential of tumor suppression in vivo was further investigated using a subcutaneous xenograft OS mouse model, building upon the promising anti-tumor performance of TCP-PLA/GeSe scaffolds observed in vitro. However, using an in situ osteosarcoma mouse model was deemed unsuitable due to inherent challenges and technical limitations [59]. In clinical osteosarcoma management, the initial step involves surgical excision of the localized tumor, followed by implantation of a bone repair scaffold material into the resultant bone defect. This sequential approach prioritizes complete tumor eradication before addressing the bone defect and promoting subsequent bone regeneration with the scaffold material. Notably, this surgical approach may not be suitable for naked mice with xenograft osteosarcoma. The unique properties of the naked mice model and the nature of xenograft tumors may necessitate alternative experimental approaches to investigating and treating osteosarcoma in this specific setting. Therefore, our study employed both an ectopic osteosarcoma model and a New Zealand rabbit radial bone defect model to validate the tumor ablation and large segment bone regeneration capabilities of TCP-PLA/GeSe scaffolds. These distinct yet interconnected models collectively provide a comprehensive assessment of the multifunctional attributes of TCP-PLA/GeSe scaffolds. By utilizing both the ectopic osteosarcoma model and the New Zealand rabbit radial bone defect model, our study directly assesses and demonstrates the diverse functionalities of TCP-PLA/GeSe scaffolds, in terms of tumor ablation and regeneration of large-segment bone defects. This approach ensures a more comprehensive understanding of the scaffold’s potential applications and therapeutic benefits.

The mice bearing subcutaneous tumors were randomly assigned to one of four treatment groups (*n* = 3): TCP-PLA, TCP-PLA/GeSe, TCP-PLA + NIR, and TCP-PLA/GeSe + NIR. Photonic tumor hyperthermia (808 nm 1.0 W/cm^2^) was conducted in the TCP-PLA + NIR and TCP-PLA/GeSe + NIR groups 1 day after scaffold implantation into the tumor site. Notably, the tumor temperature in the TCP-PLA/GeSe + NIR group rapidly increased from 29 to 55 °C within 2 min (Fig. 7E). In stark contrast, tumor temperature exhibited only a minimal increase following TCP-PLA scaffold implantation without the PLA/GeSe nanofiber membrane coating. After two weeks of treatment, the mice were sacrificed, and tumors were excised and photographed. The combination of tumor volume statistical analysis (Fig. 7F) and visual examination indicated nearly complete elimination of tumors in the TCP-PLA/GeSe + NIR group due to effective photon-induced hyperthermia (Fig. 7I,J). Although photon hyperthermia was not available in the TCP-PLA/GeSe group, tumors in this group exhibited partial inhibition, whereas Se released from the PLA/GeSe nanofiber film contributed to tumor growth inhibition. Comparatively, tumors in the other two groups showed negligible inhibition.

Subsequently, the excised tumors were subjected to pathological examination (Fig. 7H). Hematoxylin and eosin (H&E) staining and terminal deoxynucleotidyl transferase dUTP nick-end labeling (TUNEL) staining revealed increased cellular debris and cell death in tumors from the TCP-PLA/GeSe + NIR treated group, followed by the TCP-PLA/GeSe treated group. This aligns with effective in vivo tumor inhibition. Tumor cell proliferation was assessed using Ki-67 staining, demonstrating the lowest proliferation in the PLA/GeSe + NIR group, indicated by the absence of dark brown staining. This finding implies significant suppression of tumor cell proliferation in the TCP-PLA/GeSe + NIR group compared to other groups. These findings are consistent with H&E staining and TUNEL assay results. Additionally, there were no significant differences in body weights among the groups (Fig. 7G), indicating that neither TCP-PLA nor TCP-PLA/GeSe exerted significant toxicity. Comparable body weights suggest that the scaffolds were well-tolerated in vivo and did not adversely affect overall growth and development. Furthermore, the in vivo biocompatibility of TCP-PLA/GeSe scaffolds was evaluated. Histocompatibility assessment involved H&E staining of major organs, including the heart, liver, spleen, lung, and kidney. Pathological analysis revealed no significant histomorphology or pathological change in any group, underscoring the good biocompatibility of both TCP and TCP-PLA/GeSe scaffold materials in vivo (Figure S8B, Supporting Information).

## Conclusion

In summary, we have introduced a piezoelectric biomimetic bone-periosteum therapeutic platform for spatiotemporal regulated innervated bone regeneration and therapy of osteosarcoma through the construction of PLA/GeSe nanofiber membrane-coated bioceramic TCP scaffold. Under ultrasound excitation, TCP-PLA/GeSe scaffold promoted neurogenic differentiation of SCs by activating calcium signaling pathways, PI3K-AKT and Ras signaling pathways, satisfying early innervation during bone formation, and also displayed outstanding osteogenic differentiation capabilities in BMSCs. This is complemented by the porous structure of bioceramics and the sustained release of calcium and phosphorus. The in vivo evaluation of rabbit radius critical bone defect model further confirmed that the implanted TCP-PLA/GeSe scaffold enhanced early innervation and osteogenesis. Furthermore, experiments conducted in vivo and in vitro reveal that the comprehensive osteosarcoma treatment capabilities stem from the combined effect of the TCP-PLA/GeSe scaffold’s photothermal ablation potential, facilitated by the excellent photothermal properties of GeSe nanosheets, along with the sustained release of the anti-tumor element selenium. Specifically, this scaffold offers a promising solution for cases where surgically resected osteosarcoma leaves behind large segment bone defects. Our current strategy provides novel insights on the development of multifunctional biomaterials for the treatment of osteosarcoma with large segment bone defect that combine spatiotemporal regulated innervated bone regeneration with photothermal-ion therapy for osteosarcoma.

## Experimental section

### GeSe nanosheets preparation

The bulk GeSe crystals were syntheised from commercially available GeSe powders via using a chemical vapor transport method in a two-zone tube furnace [60]. GeSe nanosheets were subsequently prepared using a modified liquid exfoliation technique. GeSe crystals (20 mg) were ground into a powder and dispersed in 20 mL of absolute ethanol. The GeSe powder-containing solution was sonicated in an ice bath for 10 h and then centrifuged for 10 min at 3000 rpm. The resulting supernatant containing GeSe nanosheets was further centrifuged at 8000 rpm for 10 min to isolate the nanosheets from the precipitate. The collected precipitates were then dispersed in a solution for subsequent experiments.

### Characterization of GeSe nanosheets

SEM and TEM images of GeSe nanosheets were obtained using Nova Nano SEM 450 (FEI, The Netherlands) and Talos F200X (FEI, The Netherlands). X-ray diffraction patterns and Raman spectra of GeSe nanosheets were captured using the X’Pert-3 X-ray diffractometer and the LabRAM HR800 micro spectrometer.

GeSe nanosheets were dispersed in a PBS solution, and their photothermal conversion performance was evaluated in vitro using a NIR laser (Changchun New Industries Optoelectronics Tech. Co., Ltd) with varying power densities (0.5 W/cm^2^, 1.0 W/cm^2^, 1.5 W/cm^2,^ and 2.0 W/cm^2^).

The efficiency of the photothermal conversion of GeSe nanosheets was calculated following a methodology consistent with a previous study [61].

### Preparation of PLA/GeSe nanofiber membrane and TCP-PLA/GeSe scaffolds

A mixture of 1 g of TCP powder, 0.6 g of large pore-forming agent (NH_4_Cl powder, diameter: 150-200 μm), and 0.4 g of small pore-forming agent (NH_4_Cl powder, diameter: 60-100 μm) was pressed into a cylindrical shape (diameter: 0.5 cm, length: 1.5 cm) and sintered in a tube furnace to obtain the TCP scaffold material.

To prepare a 12.5 wt % PLA solution, 1.25 g of PLA powder was stirred in 10 mL of hexafluoroisopropanol (HFIP). GeSe nanosheets were then incorporated into the PLA solution to form mixtures with concentrations of 5%, 10%, and 20% PLA/GeSe. An electrospinning technique was used to fabricate electrospun membranes. The PLA/GeSe spinning dope was loaded in a syringe with a 21- gauge stainless steel needle. Electrospinning was implemented with a high voltage of 12 kV (ET-X1, Yongkang, Beijing) at a flow speed of 0.6 mm min-1, and a speed (3000 rpm) collecting drum with tin foil was used to keep 15 cm from the spinneret to collect the fibers. The TCP-PLA/GeSe scaffold, consisting of a PLA/GeSe nanofiber membrane covering the TCP bioceramic, was prepared through this process.

### Characterization of PLA/GeSe nanofiber membrane and TCP-PLA/GeSe scaffolds

The morphology of the PLA/GeSe nanofiber membrane and TCP-PLA/GeSe scaffolds was investigated using SEM (FEI, The Netherlands). Changes in output voltage and currents of PLA/GeSe under the US were detected using a digital storage oscilloscope (RTM3000, Germany) and a current amplifier (CX3300, United States).

### Ultrasound treatment

 Homemade US excitation equipment was composed of a commercial transducer (DYW-1 M, China), a function generator (AFG3021C, United States), and a power amplifier (1040 L, United States). Ultrasound parameters were as follows: ultrasound frequency of 100 kHz, pulse width of 50 µs, a pulse interval of 10 ms, and ultrasonic intensity of 0.7 W/cm^2^. Ultrasonic treatment was applied to cells for 20 min daily at 37 °C. Similarly, ultrasonic treatment was applied to the defect site of New Zealand rabbits after surgery for 20 min daily for one month.

### Cell culture

Rat SCs and PC12 cells were cultured in Dulbecco’s modified Eagle’s medium (DMEM) supplemented with 10% FBS at 37 °C in a humid environment with 5% CO_2_ (China Centre for Type Culture Collection, Wuhan, China). Primary BMSCs were cultured in α-MEM medium (Gibco), with 10% FBS and 100 µg/mL penicillin/streptomycin at 37 °C in a humid incubator with 5% CO_2_. Saos-2 cells were cultured in McCoy’5 A medium containing 10% foetal bovine serum (FBS, HyClone) and 1% penicillin/streptomycin at 37 °C in a humid incubator with 5% CO_2_.

### Cell viability assay

The viability of BMSCs, SCs, and Saos-2 cells on the scaffold was assessed using CCK-8 tests, calcein-AM, and propidium iodide staining at specific time points according to the manufacturer’s instructions.

### RT-PCR

Total RNA was extracted using TRIzol from cells inoculated on the hydrogels at specific time points (day 14 for BMSCs and day 5 for SCs). Gene expression was evaluated using RT-PCR. Primer sequences used in this experiment are listed in Table S1, Supporting Information.

### Differentiation of PC12 cells

Supernatants from SCs cultured on various scaffolds were collected and combined with DMEM at a 1:1 volume ratio to create SC-conditioned media. The DMEM was supplemented with 10% FBS but lacked neurotrophic factors. PC12 cells were cultured in the sc-conditioned medium for 5 days and subsequently fixed with paraformaldehyde for further assessment. To evaluate PC12 cell differentiation, the cytoskeleton was stained with rhodamine fluorescein, and nuclei were stained with DAPI, followed by observation using a fluorescence microscope. Neurite lengths of PC12 cells were calculated using ImageJ software.

### ALP and ARS staining

BMSCs were cultured under different conditions to assess their osteogenic differentiation potential. After a predetermined incubation period, the BMSCs were fixed with paraformaldehyde, washed, and stained using an ALP kit (Beyotime Institute of Biotechnology, China). Alizarin Red S (Solarbio, China) was used for AR staining.

### Immunofluorescent staining of SCs and BMSCs

Immunofluorescence staining was used to determine specific protein expression in SCs and BMSCs. Cells were fixed with paraformaldehyde, permeabilized with 0.1% Triton X-100 PBS, and blocked with 0.5% bovine serum albumin. The cells were then incubated overnight at 4 °C with primary antibodies (BGDF and S100 for SCs, OPN, and Runx-2 for BMSCs). Following that, the cytoskeleton was stained with rhodamine fluorescein and the nuclei were stained with DAPI. Fluorescence microscopy was used to assess specific protein expression.

### Large segment bone defect in new Zealand rabbits model

Four-month-old New Zealand rabbits weighing approximately 2.5 kg were used for this study. All animal experiments were approved by the Animal Committee of Tongji Medical College, Huazhong University of Science and Technology. After anesthetizing the animals, the forelimbs were sterilized, and a 15 mm-long segmental bone defect was created in the middle-upper segment of the radius after incising the skin and muscles. TCP-PLA and TCP-PLA/GeSe scaffolds were implanted into the defects. During the indicated period, the radius defects were exposed to US treatment (frequency 100 kHz, pulse width 50 µs, pulse interval 10 ms, ultrasonic intensity 0.7 W/cm^2^) for 20 min daily for 1 month. Rabbits were sacrificed after 6 and 12 weeks, and radius specimens were obtained for further analysis.

### Micro-CT scanning

At 6- and 12-weeks following surgery, bone samples were collected from the animals. Micro-CT (SkyScan 1176, Bruker, Germany) was used to calculate bone volume fraction (BV/TV) and bone mineral density (BMD) to evaluate each group’s capacity for bone healing.

### Immunofluorescence staining of tissue sections

Tissue sections obtained from bone samples were subjected to H&E staining, Masson’s trichrome staining, immunofluorescence, and immunohistochemistry. Immunofluorescence staining against OCN, OPN, Neurofilament, and CGRP was performed. Confocal laser-scanning microscopy was used to visualize the images, which were subsequently analyzed using Image J.

### In vitro photothermal performance of TCP-PLA/GeSe scaffold

TCP-PLA/GeSe scaffolds in 48-well plates were exposed to 808 nm laser radiation at varying power levels. Additionally, different concentrations of PLA/GeSe nanofiber membranes were tested. An IR thermal imaging camera was used to monitor the temperature of TCP-PLA/GeSe scaffolds. Photo-stability of the TCP-PLA/GeSe scaffolds was assessed under a power density of 1 W/cm^2^.

### In vitro photothermal ablation of osteosarcoma cells

Saos-2 cells were seeded on scaffolds and irradiated with a NIR laser. CCK-8 assay and dead-live staining were performed to assess the in vitro photothermal treatment efficacy of TCP-PLA/GeSe.

### In vivo tumor therapy with TCP-PLA/GeSe scaffolds

Female Balb/c nude mice were used for this study. Osteosarcoma was induced by subcutaneously injecting 5 × 10^6^ Saos-2 cells suspended in PBS into the mice’s right hind. In vivo PTT was performed when tumor volume reached approximately 100 mm^3^. The scaffolds were inserted into the tumor centre following a skin incision made at the tumor’s edge. After that, the wound was closed using surgical sutures. After 24 h, the mice were sedated and exposed to an 808 nm laser (1.0 W/cm^2^) for 6 min. Tumour volume was measured daily for one week following the corresponding treatments. An IR thermal imaging camera was used to monitor in-situ thermal images and real-time temperature at the mice’s tumor sites. Every two days, the body weights and tumour volumes of each mouse were recorded. Representative tumors from each treatment group underwent hematoxylin and eosin (H&E) staining, terminal deoxynucleotidyl transferase-mediated dUTP-biotin nick end labelling (TUNEL), and Ki-67 antibody staining analysis. On the fourteenth day, the major organs of mice from different groups were excised and subjected to H&E staining to further confirm the in vivo biosafety of scaffold implantation.

### Statistical analysis

The data were analyzed using GraphPad Prism (version 7.0). One-way analysis of variance was used for statistical analysis, followed by Tukey’s multiple comparisons tests. The sample size (n) for each experiment is provided in the figure legends. *p* < 0.05 was considered statistically significant.

### Electronic supplementary material

Below is the link to the electronic supplementary material.

Supplementary Material 1

## Data Availability

The data that support the findings of this study are available from the corresponding author upon reasonable request.

## References

[1] Liu K, Liao Y, Zhou Z, Zhang L, Jiang Y, Lu H, Xu T, Yang D, Gao Q, Li Z, Tan S, Cao W, Chen F, Li G (2022). Photothermal-triggered immunogenic nanotherapeutics for optimizing osteosarcoma therapy by synergizing innate and adaptive immunity. Biomaterials.

[2] Zhao Y, Peng X, Xu X, Wu M, Sun F, Xin Q, Zhang H, Zuo L, Cao Y, Xia Y, Luo J, Ding C, Li J (2023). Chitosan based photothermal scaffold fighting against bone tumor-related complications: recurrence, infection, and defects. Carbohydr Polym.

[3] Chu X, Zhang L, Li Y, He Y, Zhang Y, Du C (2023). NIR Responsive Doxorubicin-Loaded Hollow Copper Ferrite @ Polydopamine for synergistic Chemodynamic/Photothermal/Chemo-Therapy. Small.

[4] Yang Q, Yin H, Xu T, Zhu D, Yin J, Chen Y, Yu X, Gao J, Zhang C, Chen Y, Gao Y (2020). Engineering 2D Mesoporous Silica@MXene-Integrated 3D-Printing scaffolds for Combinatory Osteosarcoma Therapy and NO-Augmented bone regeneration. Small.

[5] Marrella A, Lee TY, Lee DH, Karuthedom S, Syla D, Chawla A, Khademhosseini A, Jang HL (2018). Engineering vascularized and innervated bone biomaterials for improved skeletal tissue regeneration. Mater Today (Kidlington).

[6] Wang X, Li S, Zhang S, Gupta A, Zhang C, Wang L (2020). The neural system regulates bone homeostasis via mesenchymal stem cells: a translational approach. Theranostics.

[7] Zhang Z, Wang F, Huang X, Sun H, Xu J, Qu H, Yan X, Shi W, Teng W, Jin X, Shao Z, Zhang Y, Zhao S, Wu Y, Ye Z, Yu X (2023). Engineered sensory nerve guides Self-Adaptive Bone Healing via NGF-TrkA signaling pathway. Adv Sci (Weinh).

[8] Ma YX, Jiao K, Wan QQ, Li J, Liu MY, Zhang ZB, Qin W, Wang KY, Wang YZ, Tay FR, Niu LN (2022). Silicified collagen scaffold induces semaphorin 3A secretion by sensory nerves to improve in-situ bone regeneration. Bioact Mater.

[9] Li Z, Meyers CA, Chang L, Lee S, Li Z, Tomlinson R, Hoke A, Clemens TL (2019). James A W. Fracture repair requires TrkA signaling by skeletal sensory nerves. J Clin Investig.

[10] Leitão L, Neto E, Conceição F, Monteiro A, Couto M, Alves CJ, Sousa DM, Lamghari M (2020). Osteoblasts are inherently programmed to repel sensory innervation. Bone Res.

[11] Tomlinson RE, Li Z, Zhang Q, Goh BC, Li Z, Thorek D, Rajbhandari L, Brushart TM, Minichiello L, Zhou F, Venkatesan A, Clemens TL (2016). NGF-TrkA signaling by sensory nerves coordinates the vascularization and ossification of developing endochondral bone. Cell Rep.

[12] Tomlinson RE, Li Z, Li Z, Minichiello L, Riddle RC, Venkatesan A, Clemens TL (2017). NGF-TrkA signaling in sensory nerves is required for skeletal adaptation to mechanical loads in mice. Proc Natl Acad Sci U S a.

[13] Zhang H, Zhang M, Zhai D, Qin C, Wang Y, Ma J, Zhuang H, Shi Z, Wang L, Wu C. Polyhedron-Like Biomaterials for Innervated and Vascularized Bone Regeneration. Adv Mater. 2023:e2302716. 10.1002/adma.202302716 37434296

[14] Yu Y, Wang Y, Zhang W, Wang H, Li J, Pan L, Han F, Li B (2020). Biomimetic periosteum-bone substitute composed of preosteoblast-derived matrix and hydrogel for large segmental bone defect repair. Acta Biomater.

[15] Zhang D, Tan J, Xu R, Du H, Xie J, Peng F, Liu X (2023). Collaborative design of MgO/FeO(x) nanosheets on Titanium: combining therapy with regeneration. Small.

[16] Zhang H, Qin C, Zhang M, Han Y, Ma J, Wu J, Yao Q, Wu C (2022). Calcium silicate nanowires-containing multicellular bioinks for 3D bioprinting of neural-bone constructs. Nano Today.

[17] Li W, Miao W, Liu Y, Wang T, Zhang Y, Wang W, Lu D, Zhou X, Jiao X, Jia X (2022). Bioprinted constructs that mimic the ossification center microenvironment for targeted innervation in bone regeneration. Adv Funct Mater.

[18] Wang L, Pang Y, Tang Y, Wang X, Zhang D, Zhang X, Yu Y, Yang X, Cai Q (2023). A biomimetic piezoelectric scaffold with sustained Mg2 + release promotes neurogenic and angiogenic differentiation for enhanced bone regeneration. Bioact Mater.

[19] Xu C, Chang Y, Wu P, Liu K, Dong X, Nie A, Mu C, Liu Z, Dai H, Luo Z (2021). Two-dimensional‐Germanium Phosphide‐Reinforced Conductive and Biodegradable Hydrogel scaffolds enhance spinal cord Injury Repair. Adv Funct Mater.

[20] Xu Y, Xu C, He L, Zhou J, Chen T, Ouyang L, Guo X, Qu Y, Luo Z, Duan D (2022). Stratified-structural hydrogel incorporated with magnesium-ion-modified black phosphorus nanosheets for promoting neuro-vascularized bone regeneration. Bioact Mater.

[21] Gong B, Zhang X, Zahrani AA, Gao W, Ma G, Zhang L, Xue J (2022). Neural tissue engineering: from bioactive scaffolds and in situ monitoring to regeneration. Explor (Beijing).

[22] Chen P, Xu C, Wu P, Liu K, Chen F, Chen Y, Dai H, Luo Z (2022). Wirelessly powered Electrical-Stimulation based on biodegradable 3D Piezoelectric Scaffolds promotes the spinal cord Injury Repair. ACS Nano.

[23] Tan MH, Xu XH, Yuan TJ, Hou X, Wang J, Jiang ZH, Peng LH (2022). Self-powered smart patch promotes skin nerve regeneration and sensation restoration by delivering biological-electrical signals in program. Biomaterials.

[24] Wan X, Zhao Y, Li Z, Li L (2022). Emerging polymeric electrospun fibers: from structural diversity to application in flexible bioelectronics and tissue engineering. Explor (Beijing).

[25] Varga M, Morvan J, Diorio N, Buyuktanir E, Harden J, West JL, Jákli A (2013). Direct piezoelectric responses of soft composite fiber mats. Appl Phys Lett.

[26] Ribeiro HB, Ramos S, Seixas L, De Matos C, Pimenta MA (2019). Edge phonons in layered orthorhombic GeS and GeSe monochalcogenides. Phys Rev B.

[27] Zhai W, Lai Q, Chen L, Zhu L, Wang ZL (2020). Flexible piezoelectric nanogenerators based on P (VDF–TrFE)/GeSe nanocomposite films. Acs Appl Electron Mater.

[28] Chai Z, Zhang W, Clima S, Hatem F, Degraeve R, Diao Q, Zhang JF, Freitas P, Marsland J, Fantini A (2021). Cycling induced metastable degradation in GeSe Ovonic threshold switching selector. Ieee Electron Device Lett.

[29] Li X, Wang Y, Chen Y, Zhou P, Wei K, Wang H, Wang J, Fang H, Zhang S (2020). Hierarchically constructed selenium-doped bone-mimetic nanoparticles promote ROS-mediated autophagy and apoptosis for bone tumor inhibition. Biomaterials.

[30] Wang Y, Hao H, Liu H, Wang Y, Li Y, Yang G, Ma J, Mao C, Zhang S (2015). Selenite-releasing bone Mineral nanoparticles Retard Bone Tumor Growth and improve healthy tissue functions in vivo. Adv Healthc Mater.

[31] Liu SC, Mi Y, Xue DJ, Chen YX, He C, Liu X, Hu JS, Wan LJ (2017). Investigation of physical and electronic properties of GeSe for photovoltaic applications. Adv Electron Mater.

[32] Duan C, Yu M, Xu J, Li B, Zhao Y, Kankala RK (2023). Overcoming Cancer Multi-drug Resistance (MDR): reasons, mechanisms, nanotherapeutic solutions, and challenges. Biomed Pharmacother.

[33] Bian D, Zhou W, Deng J, Liu Y, Li W, Chu X, Xiu P, Cai H, Kou Y, Jiang B, Zheng Y (2017). Development of magnesium-based biodegradable metals with dietary trace element germanium as orthopaedic implant applications. Acta Biomater.

[34] Zhou N, Long H, Wang C, Yu L, Zhao M, Liu X (2020). Research progress on the biological activities of selenium polysaccharides. Food Funct.

[35] Dang W, Yi K, Ju E, Jin Y, Xu Y, Wang H, Chen WC, Wang K, Wang Y, Tao Y, Li M (2021). 3D printed Bioceramic scaffolds as a Universal Therapeutic platform for synergistic therapy of Osteosarcoma. Acs Appl Mater Interfaces.

[36] Xu C, Xia Y, Zhuang P, Liu W, Mu C, Liu Z, Wang J, Chen L, Dai H, Luo Z (2023). FePSe3-Nanosheets-Integrated Cryogenic-3D-Printed multifunctional calcium phosphate scaffolds for synergistic therapy of Osteosarcoma. Small.

[37] Han Y, Liu C, Chen B, Fu C, Kankala RK, Wang S, Chen A (2022). Orchestrated tumor apoptosis (Cu2+) and bone tissue calcification (Ca2+) by hierarchical Copper/Calcium-ensembled bioactive silica for osteosarcoma therapy. Chem Eng J.

[38] Ren K, Zhu M, Song W, Lv S, Xia M, Yong W, Lu Y, Ji Z, Song Z (2018). Electrical switching properties and structural characteristics of GeSe-GeTe films. Nanoscale.

[39] Yap WC, Yang Z, Mehboudi M, Yan J, Barraza-Lopez S, Zhu W (2018). Layered material GeSe and vertical GeSe/MoS2 p-n heterojunctions. Nano Res.

[40] Zeng J, Goldfeld D, Xia Y (2013). A Plasmon-assisted optofluidic (PAOF) system for measuring the Photothermal Conversion Efficiencies of Gold Nanostructures and Controlling an Electrical switch. Angew Chem Int Ed.

[41] Manikandan M, Hasan N, Wu H (2013). Platinum nanoparticles for the photothermal treatment of Neuro 2A cancer cells. Biomaterials.

[42] Perez RA, Mestres G (2016). Role of pore size and morphology in musculo-skeletal tissue regeneration. Mater Sci Eng C Mater Biol Appl.

[43] Pérez-Hernández M, Del PP, Mitchell SG, Moros M, Stepien G, Pelaz B, Parak WJ, Gálvez EM, Pardo J, de la Fuente JM (2015). Dissecting the molecular mechanism of apoptosis during photothermal therapy using gold nanoprisms. ACS Nano.

[44] Fan T, Yan L, He S, Hong Q, Ai F, He S, Ji T, Hu X, Ha E, Zhang B, Li Z, Zhang H, Chen X, Hu J (2022). Biodistribution, degradability and clearance of 2D materials for their biomedical applications. Chem Soc Rev.

[45] Chen L, Chen C, Chen W, Li K, Chen X, Tang X, Xie G, Luo X, Wang X, Liang H, Yu S (2018). Biodegradable black phosphorus nanosheets mediate specific delivery of hTERT siRNA for synergistic Cancer therapy. Acs Appl Mater Interfaces.

[46] Rao F, Wang Y, Zhang D, Lu C, Cao Z, Sui J, Wu M, Zhang Y, Pi W, Wang B, Kou Y, Wang X, Zhang P, Jiang B (2020). Aligned chitosan nanofiber hydrogel grafted with peptides mimicking bioactive brain-derived neurotrophic factor and vascular endothelial growth factor repair long-distance sciatic nerve defects in rats. Theranostics.

[47] Ning C, Zhou Z, Tan G, Zhu Y, Mao C (2018). Electroactive polymers for tissue regeneration: developments and perspectives. Prog Polym Sci.

[48] Tang G, Dong X, Huang X, Huang XJ, Liu H, Wang Y, Ye WC, Shi L (2015). A natural diarylheptanoid promotes neuronal differentiation via activating ERK and PI3K-Akt dependent pathways. Neuroscience.

[49] Zhong J (2016). RAS and downstream RAF-MEK and PI3K-AKT signaling in neuronal development, function and dysfunction. Biol Chem.

[50] Ghosh A, Greenberg ME (1995). Calcium signaling in neurons: molecular mechanisms and cellular consequences. Science.

[51] Qian Y, Zhao X, Han Q, Chen W, Li H, Yuan W (2018). An integrated multi-layer 3D-fabrication of PDA/RGD coated graphene loaded PCL nanoscaffold for peripheral nerve restoration. Nat Commun.

[52] Salhotra A, Shah HN, Levi B, Longaker MT (2020). Mechanisms of bone development and repair. Nat Rev Mol Cell Biol.

[53] Jones RE, Salhotra A, Robertson KS, Ransom RC, Foster DS, Shah HN, Quarto N, Wan DC, Longaker MT (2019). Skeletal stem cell-Schwann Cell Circuitry in Mandibular Repair. Cell Rep.

[54] Wan QQ, Qin WP, Ma YX, Shen MJ, Li J, Zhang ZB, Chen JH, Tay FR, Niu LN, Jiao K (2021). Crosstalk between bone and nerves within bone. Adv Sci (Weinh).

[55] Khalil M, Teunissen CE, Otto M, Piehl F, Sormani MP, Gattringer T, Barro C, Kappos L, Comabella M, Fazekas F, Petzold A, Blennow K, Zetterberg H, Kuhle J (2018). Neurofilaments as biomarkers in neurological disorders. Nat Rev Neurol.

[56] Mi J, Xu J, Yao Z, Yao H, Li Y, He X, Dai B, Zou L, Tong W, Zhang X, Hu P, Ruan YC, Tang N, Guo X, Zhao J, He J, Qin L (2022). Implantable Electrical stimulation at dorsal Root ganglions accelerates osteoporotic fracture Healing via Calcitonin Gene-related peptide. Adv Sci (Weinh).

[57] Ye L, Xu J, Mi J, He X, Pan Q, Zheng L, Zu H, Chen Z, Dai B, Li X, Pang Q, Zou L, Zhou L, Huang L, Tong W, Li G, Qin L (2021). Biodegradable magnesium combined with distraction osteogenesis synergistically stimulates bone tissue regeneration via CGRP-FAK-VEGF signaling axis. Biomaterials.

[58] Xu C, Xia Y, Zhuang P, Liu W, Mu C, Liu Z, Wang J, Chen L, Dai H, Luo Z. FePSe(3) -Nanosheets-Integrated Cryogenic-3D-Printed multifunctional calcium phosphate scaffolds for synergistic therapy of Osteosarcoma. Small. 2023:e2303636. 10.1002/smll.202303636 37217971

[59] Blattmann C, Thiemann M, Stenzinger A, Roth EK, Dittmar A, Witt H, Lehner B, Renker E, Jugold M, Eichwald V, Weichert W, Huber PE, Kulozik AE (2015). Establishment of a patient-derived orthotopic osteosarcoma mouse model. J Transl Med.

[60] Wang X, Li Y, Huang L, Jiang XW, Jiang L, Dong H, Wei Z, Li J, Hu W (2017). Short-Wave Near-Infrared Linear Dichroism of two-Dimensional Germanium Selenide. J Am Chem Soc.

[61] Ren X, Liu W, Zhou H, Wei J, Mu C, Wan Y, Yang X, Nie A, Liu Z, Yang X, Luo Z (2022). Biodegradable 2D GeP nanosheets with high photothermal conversion efficiency for multimodal cancer theranostics. Chem Eng J.

