# Supplementary material for: Biomimetic bone-periosteum scaffold for spatiotemporal regulated innervated bone regeneration and therapy of osteosarcoma
Source: J Nanobiotechnology. 2024 May 15;22:250. doi: 10.1186/s12951-024-02430-7 (PMC11094931; doi:10.1186/s12951-024-02430-7)
Supplement: Supplementary file 1 — Supplementary Material 1 [file 12951_2024_2430_MOESM1_ESM.docx]

Supporting Information

**Biomimetic Bone-periosteum Scaffold for Spatiotemporal Regulated Innervated Bone Regeneration and Therapy of Osteosarcoma**

*Yan Xu^#^, Chao Xu^#^, Huan Song^#^*, *Xiaobo Feng, Liang Ma, Xiaoguang Zhang, Gaocai Li, Congpu Mu, Lei Tan, Zhengdong Zhang^*^*, *Zhongyuan Liu, Zhiqiang Luo^*^, Cao Yang^*^*

Yan Xu, Xiaobo Feng, Liang Ma, Xiaoguang Zhang*,* Gaocai Li, Lei Tan, Cao Yang

Department of Orthopaedics, Union Hospital, Tongji Medical College, Huazhong University of Science and Technology, Wuhan 430074, China

E-mail: caoyangunion@hust.edu.cn

Chao Xu

College of Materials Science and Engineering, Wuhan Textile University, China Wuhan 430200, China

Huan Song

Otorhinolaryngology Head and Neck Surgery, Wuhan Fourth Hospital, Wuhan 430033, Hubei, China

Zhiqiang Luo

College of Life Science and Technology, Huazhong University of Science and Technology, China Wuhan 430022, China

E-mail: [zhiqiangluo@hust.edu.cn](mailto:zhiqiangluo@hust.edu.cn)

Zhengdong Zhang

School of Clinical Medicine, Chengdu Medical College; Department of Orthopedics, the First Affiliated Hospital of Chengdu Medical College, Chengdu 610000, China

Department of Orthopaedics, Union Hospital, Tongji Medical College, Huazhong University of Science and Technology, Wuhan 430074, China

E-mail: zhangzd@cmc.edu.cn

Congpu Mu, Zhongyuan Liu

Center for High Pressure Science, State Key Laboratory of Metastable Materials Science and Technology, Yanshan University, Qinhuangdao 066004, China


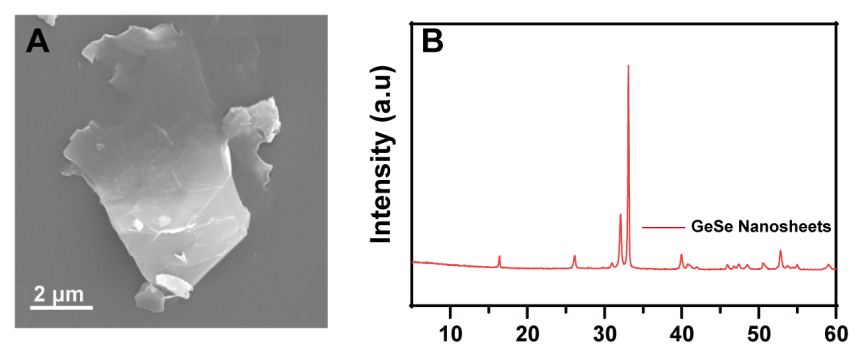


**Figure S1**. (A) SEM images of GeSe nanosheets. (B) The XRD result of GeSe nanosheets.


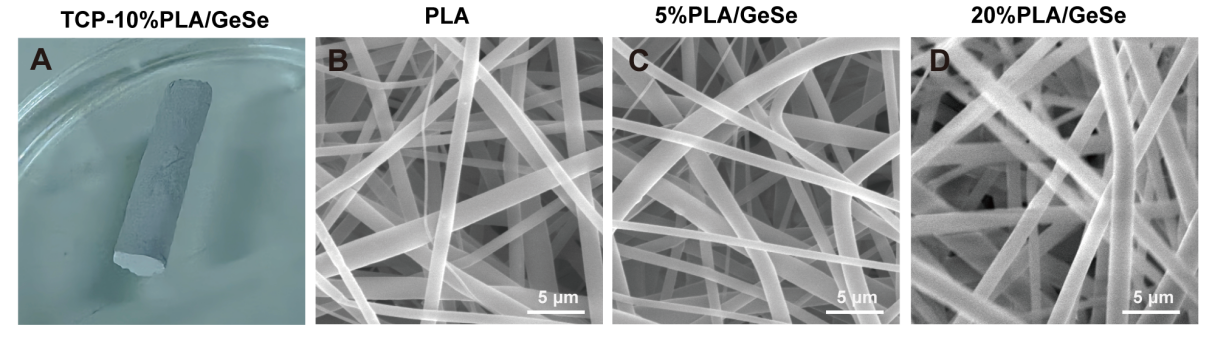


**Figure S2**. (A) General iamge of TCP-PLA/GeSe. SEM images of (B) PLA, (C) 5%PLA/GeSe, (D) 20%PLA/GeSe nanofiber membrane.


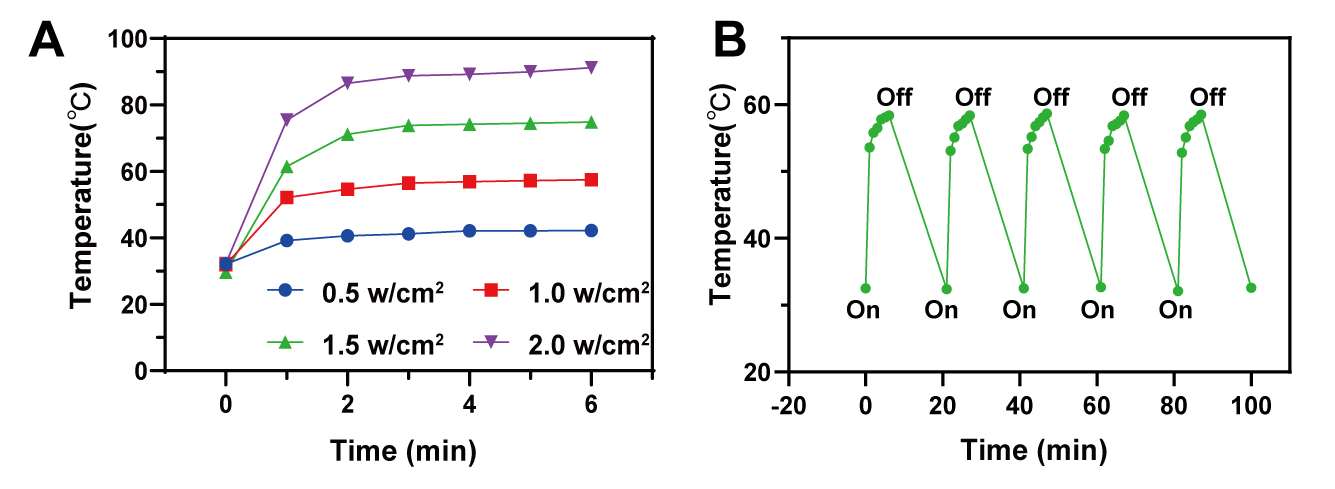


**Figure S3**. (A) Temperature changing curves of TCP-PLA/GeSe scaffold *in vitro* at different laser power densities of NIR. (B) Temperature profiles of scaffolds during four laser on/off cycles.


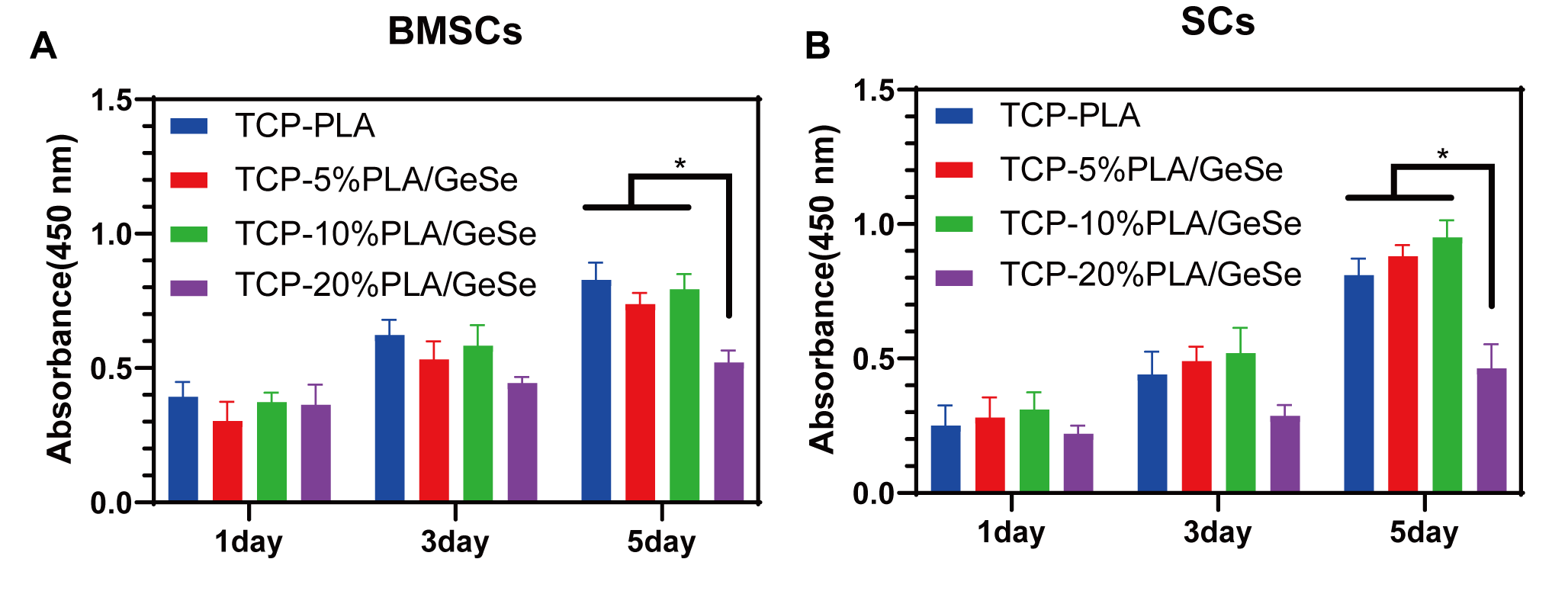


**Figure S4.** Proliferation of (A) BMSCs and (B) SCs on different scaffolds. (C) Ca, P, Ge, Se ions released from the TCP-PLA/GeSe scaffolds. n=3. A significant difference is indicated by * p < 0.05.


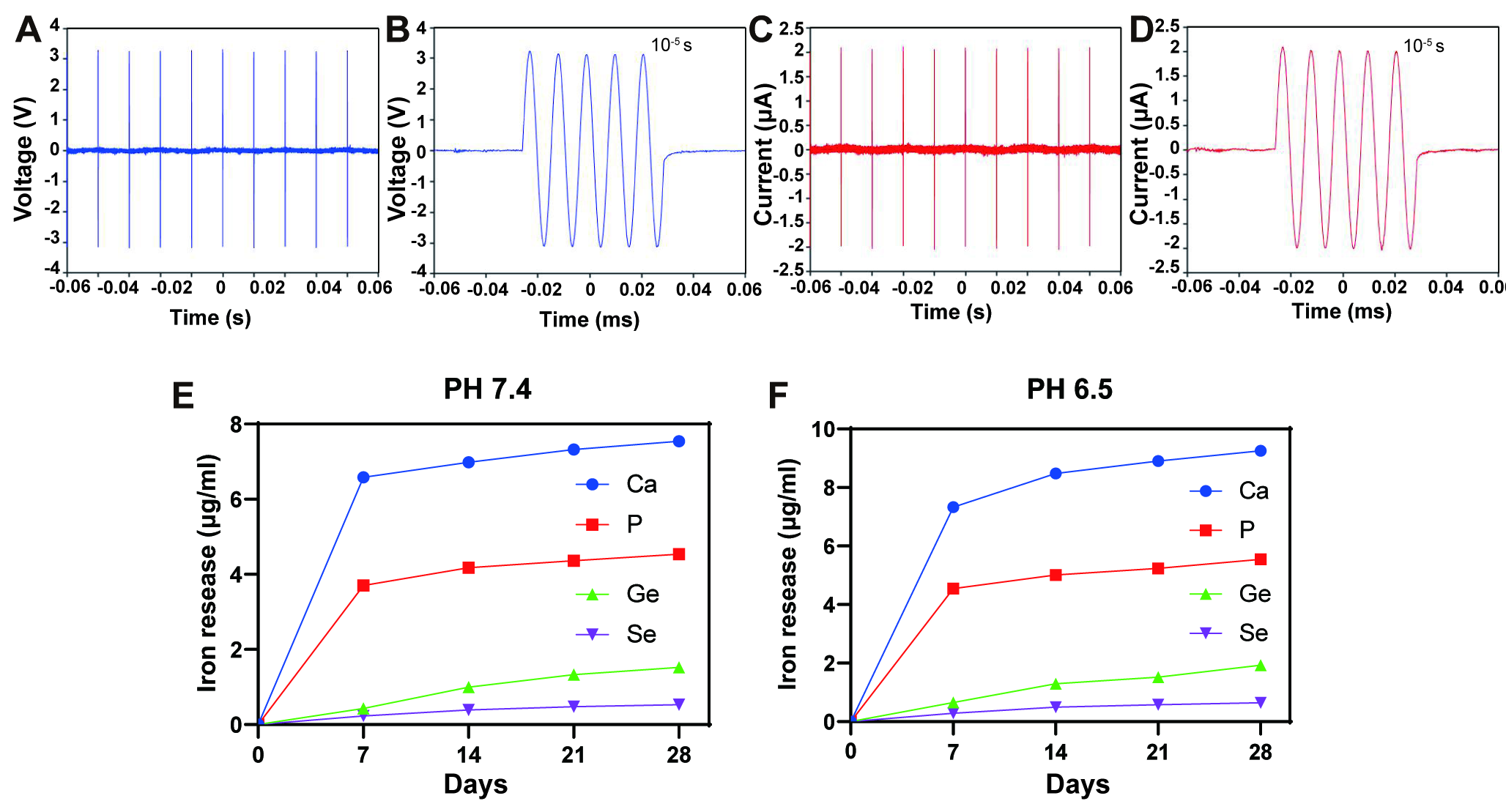


**Figure S5**. (A-B) The open-circuit voltage generated by TCP-PLA scaffolds under US excitation. (C-D) The short-circuit current generated by TCP-PLA scaffolds under US excitation. (E) Ca, P, Ge, Se ions released from the TCP-PLA/GeSe scaffolds at PH 7.4. (F) Ca, P, Ge, Se ions released from the TCP-PLA/GeSe scaffolds at PH 6.5.


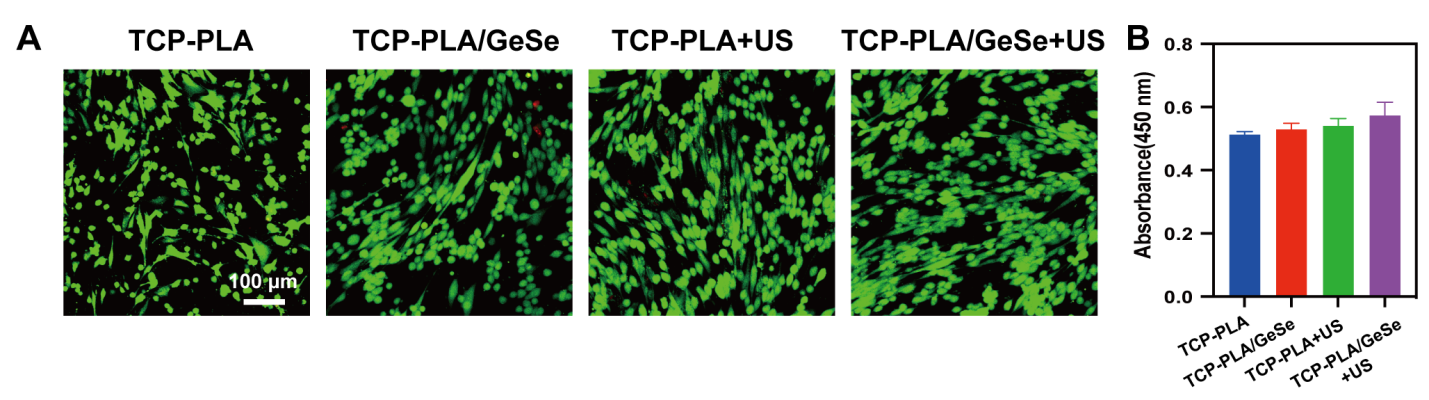


**Figure S6**. (A) Live/dead staining of SCs cultured in different groups. (B) Viability of SCs cultured on different scaffolds.


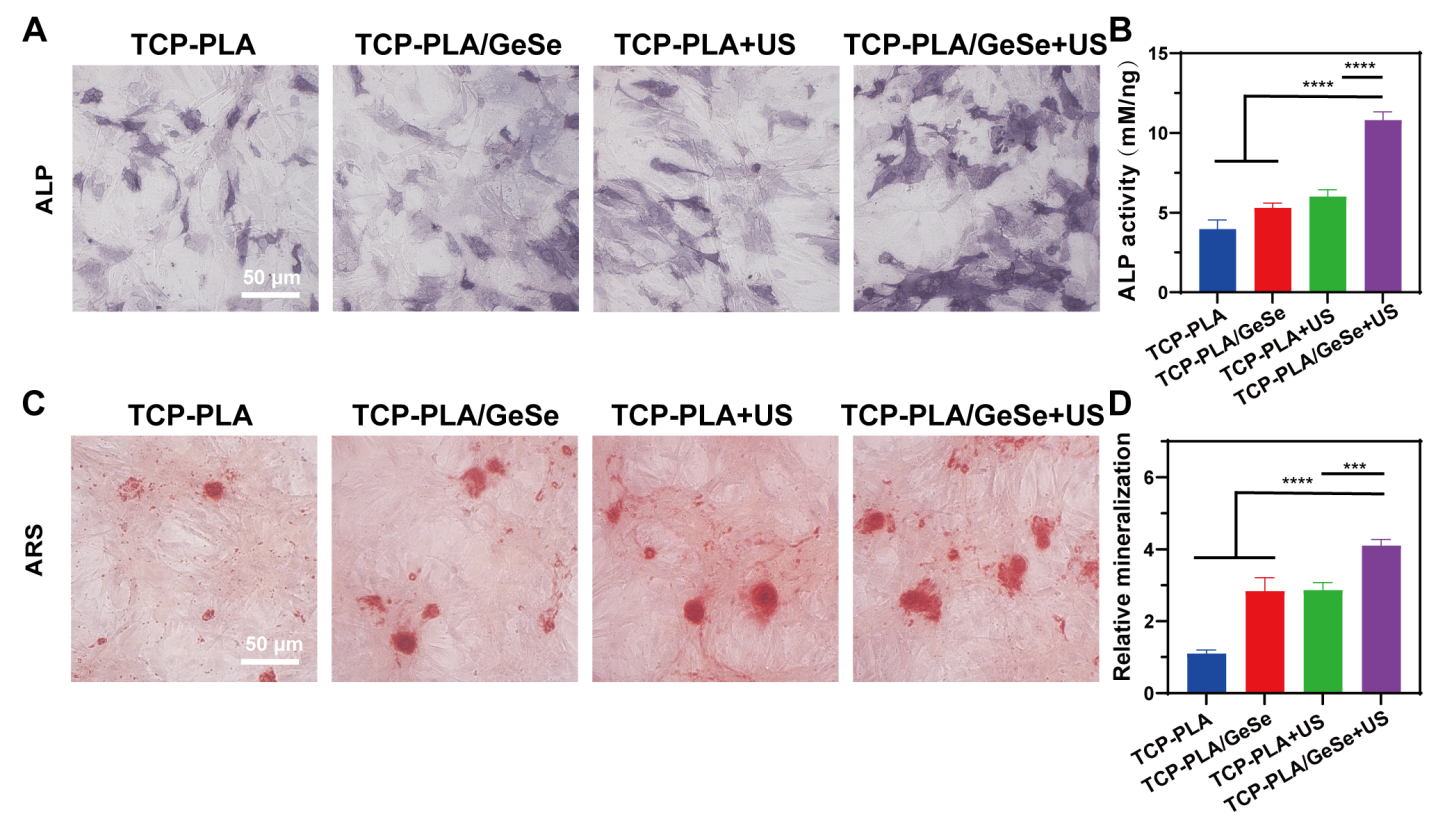


**Figure S7**. (A) ARS staining after BMSCs cultured in SC-conditioned medium of different groups. (B) ARS quantitative assay in different groups. (C) ALP staining after BMSCs cultured in SC-conditioned medium of different groups. (D) ALP quantitative assay in different groups. n=3. (* p < 0.05, ** p < 0.01, *** p < 0.001).


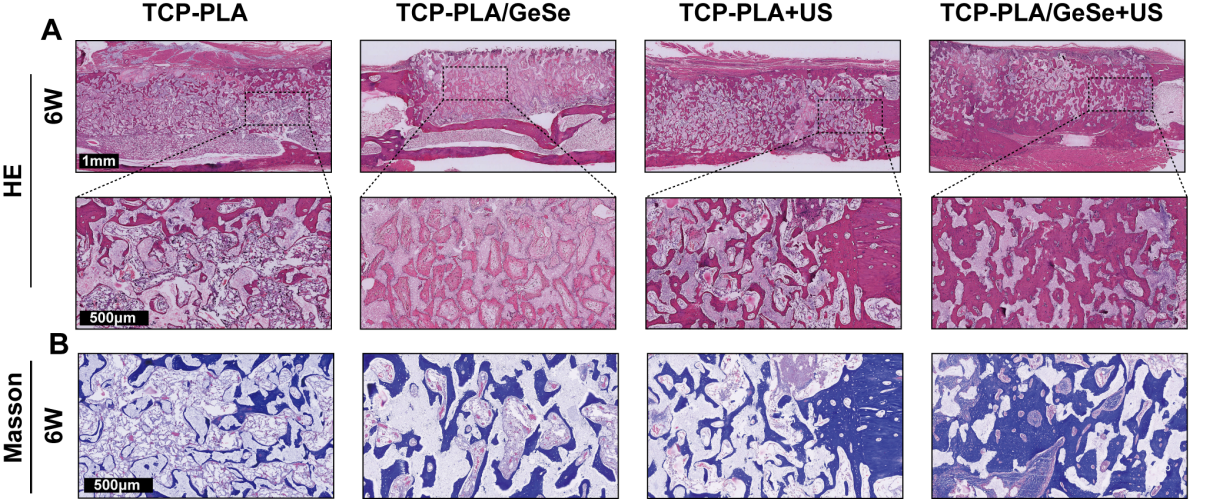


**Figure S8.** (A) H&E and (B) Masson staining at 6 weeks after surgery.


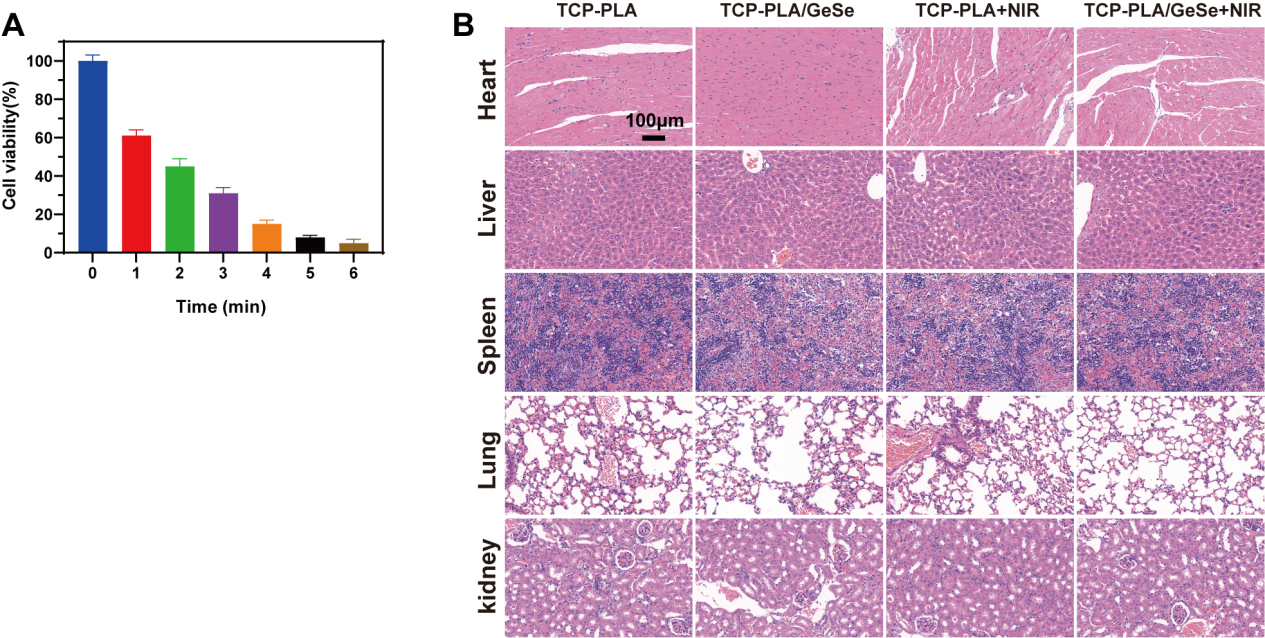


**Figure S9.** (A) The effects of laser power irradiation time on cell viability Saos-2 treated with TCP-PLA/GeSe. (B) H&E stains of heart, liver, spleen, lung and kidney of mice within different groups.

**Supplementary Table 1**. Primers for Real-Time PCR Analysis

| **Gene**  **name** | **Forward primer** | **Reverse primer** |
| --- | --- | --- |
| BDNF | GTCAAGTGCCTTTGGAGCCT | CTTATGAACCTTTGGAGCCT |
| NGF | TGATCGGCGTACAGGCAGA | GAGGGCTGTGTCAAGGGAAT |
| OPN | CCAGGTCAGAGAGGCAGAAT | GAGACCGTCTGAAACAGCGT |
| Runx-2 | TTCCTGTGCTCCGTGCTG | AAAGTGAAACTCTTGCCTCGTC |
| Col-1 | GACCTCAAGATGTGCCACT | GAACCTTCGCTTCCATACTCG |
| ALP | CGGACAATGAGATGCGCCC | TGGGAGTGCTTGTGTCTAGG |
